# Receptive Field Vectors of Genetically-Identified Retinal Ganglion Cells Reveal Cell-Type-Dependent Visual Functions

**DOI:** 10.1371/journal.pone.0147738

**Published:** 2016-02-04

**Authors:** Matthew L. Katz, Tim J. Viney, Konstantin Nikolic

**Affiliations:** 1 Centre for Bio-Inspired Technology, Institute of Biomedical Engineering, Department of Electrical and Electronic Engineering, The Bessemer Building, Imperial College London, London SW7 2AZ, United Kingdom; 2 Neural Circuit Laboratories, Friedrich Miescher Institute for Biomedical Research, 4058 Basel, Switzerland; 3 University of Basel, 4003 Basel, Switzerland; University College London, UNITED KINGDOM

## Abstract

Sensory stimuli are encoded by diverse kinds of neurons but the identities of the recorded neurons that are studied are often unknown. We explored in detail the firing patterns of eight previously defined genetically-identified retinal ganglion cell (RGC) types from a single transgenic mouse line. We first introduce a new technique of deriving receptive field vectors (RFVs) which utilises a modified form of mutual information (“Quadratic Mutual Information”). We analysed the firing patterns of RGCs during presentation of short duration (~10 second) *complex visual scenes* (natural movies). We probed the high dimensional space formed by the visual input for a much smaller dimensional subspace of RFVs that give the most information about the response of each cell. The new technique is very efficient and fast and the derivation of novel types of RFVs formed by the natural scene visual input was possible even with limited numbers of spikes per cell. This approach enabled us to estimate the 'visual memory' of each cell type and the corresponding receptive field area by calculating Mutual Information as a function of the number of frames and radius. Finally, we made predictions of biologically relevant functions based on the RFVs of each cell type. RGC class analysis was complemented with results for the cells’ response to *simple* visual input in the form of black and white spot stimulation, and their classification on several key physiological metrics. Thus RFVs lead to predictions of biological roles based on limited data and facilitate analysis of sensory-evoked spiking data from defined cell types.

## Introduction

In the mammalian retina, signals from the photoreceptors are processed by parallel neural circuits across distinct retinal layers [1, 2]. These circuits have evolved to allow the retina to effectively break down the spatio-temporal features of the visual input into parallel channels that capture different representations of the visual scene [3–5]. The exact number of different ganglion cell types in the retina is still not known [6]. The term PV retina refers to the retina of the *P**ar**v**albumin*^*Cre*^;*Thy*^*Stp-EYFP*^ mouse line in which a subpopulation of retinal ganglion cells (RGCs) express YFP [7, 8]. Using two-photon-targeted loose cell-attached recordings and whole cell patch clamp to label single cells with the marker neurobiotin, 8 distinct types of RGCs in the PV retina were identified based on *post hoc* analysis of dendritic stratification, dendritic field size, cell shape, and their responses to black/white spot visual stimulation [7].

In order to identify the visual features that the PV RGCs are sensitive to and determine their functional behaviour, retinas were presented with *natural stimuli*–sequences of movies recorded by a camera mounted to a cat's head which was freely moving in a forest [9, 10]. Experiments and qualitative analysis of the visual responses of the 8 PV RGCs to *simple* flashing spot stimuli, as reported by Farrow et al. 2013, are quantified here to complement our novel information theoretic analysis of *natural stimuli*. This *complex* type of visual input should be much more efficient in revealing the relevant receptive fields, requiring a relatively small number of inputs in comparison with white noise analysis [11, 12]. However, with such a reduced and non-Gaussian input it is not possible to use standard reverse-correlation methods to quantify the average natural stimulus that invokes a neuronal response [13–16] or its information-theoretic generalisation [17].

Two recent studies demonstrated a computational tool for studying population coding by developing model cells that mimic the responses of real RGCs [18], and showed how to use these models for retinal prosthetic applications [19]. For receptive field calculations they used a generalised spike-triggered average-based methodology proposed by Paninski et.al. [20]. Parameters for the model were determined by maximizing the likelihood that the model would produce the experimentally-observed spike trains elicited by the stimuli and demonstrated on 10x10 pixel input images. Receptive field organisation in primary visual cortex was investigated using standard reverse correlation method by Smyth et.al. [11], but they only used single static images of natural scenes, of reduced resolution (50x50 pixels), lacking the time component. A systematic study of neural coding based on information theory by de Ruyter van Steveninck and Bialek [21] introduced quantitative measures of the information transferred by sensory neurons [22]. Brenner et al [23] provided a method for calculating the average information carried by a single spike and compound patterns and compared them to deduce possible synergy in spike bursts.

Our aim was to probe the high dimensional space formed by the visual input (which is of the order of ~750,000 dimensions, corresponding to approximately ten frames at the resolution of 320x240 pixels) for a much lower dimensional subspace of receptive field vectors (RFVs). The RFVs give the most relevant information about the feature selectivity of neurons, i.e. an RGC’s local circuit. For example, in the case of a single RFV that separates spiking from non-spiking inputs, it can be interpreted as a set of illumination patterns which cause the maximum spiking response. We adopted an approach based on a form of biological “data-mining”, which depends only on the visual input that is used to stimulate the retina, and so it can be employed for any type of stimulus without requiring a model of the underlying retinal circuitry. Non-parametric models are “data dependent" with their complexity scaling to accommodate the available data, and particularly appealing for us were those based on maximally informative features [24].

A similar method has previously been developed that searches for these lower dimensional subspaces by sequentially optimising the mutual information (MI) across different RFVs [25]. While promising, this method was computationally demanding and, in their synthetic examples, used a large number of spike responses (typically >10,000 spikes) for characterisation of a visual stimulus with ~10,000 dimensions. For our experimental data, we consider a much higher dimensional stimulus space but have only a small number of responses (several hundred spikes). Hence even the improved information theoretic methods proposed in [26] are not sufficient. This has led us to adopt a related approach based on a modified form of MI known as “quadratic mutual information” (QMI), first proposed by Torkkola [27] and has been successfully implemented for machine learning algorithms [28].

The classical Shannon’s measure for entropy and the corresponding MI measure (Kullback–Leibler (KL) distance between two probability distributions, eq (1) [29]) can be substituted by a number of different measures for entropy and information when the postulate of additivity is redefined, as described by Renyi [30]. This is particularly useful when the aim is not to compute an absolute value of the entropy or a divergence measure, but rather to find a distribution that maximizes or minimizes the entropy or divergence given some constraints [31]. In our case, by maximising across the space of all possible receptive field projections the relevant RFVs of the cell can be obtained. Although this method has already been used successfully [25], it has several drawbacks in our case. Due to the complexity of calculating the MI as defined by eq (1), optimization is difficult for high dimensional stimuli and must be performed iteratively for each RFV to ensure they are orthogonal and hence it is computationally extremely demanding. In our approach we use the QMI as an objective function for optimization and selection of the RFVs [27]. QMI is a type of Renyi divergence of order 2 and provides a lower bound on KL-divergence and Shannon’s MI (based on Pinsker’s inequality) [27, 30, 31]. By using this measure we are able to perform the optimization process in search of the RFVs much more efficiently. The main advantage of using a quadratic divergence is that all integrals in eq (2) can be analytically solved if the probability distribution functions are estimated with a Parzen density method using Gaussian kernels (see Methods and ref.[32]). The process of maximising QMI will give the same RFVs as the optimization process for MI, as shown by Kapur [31], further proven by Torkkola [27] and elaborated by Principe et.al. [32].

Furthermore, use of Gaussian Parzen-windows to estimate the probability distribution functions allows direct calculation of the natural gradient of QMI as a function of the RFV transform. It has also been demonstrated that QMI gives better stability in the optimization than using other information based techniques such as MI [32]. Indeed, it was recently shown that optimization of a Rényi entropy was effective in characterizing neural feature selectivity in a simple model cell that used natural stimuli [33]. For evaluating statistical properties of the RFVs we used a jackknife method, similar to the maximally informative dimensions calculations of Rowekamp and Sharpee [34].

## Results

### Quantification of neurobiotin-labelled PV cells

Over 600 YFP-expressing ganglion cells in the PV retina were targeted with glass electrodes and their responses to visual stimulation were recorded (Table 1). 286 recorded cells are analysed here, and 183 of them were labelled with neurobiotin. 8 ganglion cell types (PV0 to PV7, S1(a) Fig) were defined based on the combination of dendritic stratification (S1(b) and S1(c) Fig, n = 182 cells from ref. [7] that includes 2 PV1 cells and 45 PV5 cells from ref. [8]), dendritic field area (S1(a) and S1(c) Fig), and spiking responses induced by different size black and white spot stimuli (n = 286 recorded cells that includes 83 cells from ref. [7]). The cell types were not classified solely on “morphological” or “physiological” properties, although now it is possible to predict the cell type by measuring single parameters, such as the spiking responses to spot stimuli or the dendritic stratification.

**Table 1:** Quantification of PV ganglion cell parameters. BS, black spot; WS, white spot; NatS, natural scene/movie. Note, a one degree visual angle corresponds to ~31 μm on the mouse retina [48]. Blue rows are OFF layers, rose are ON layers and the green coloured row (PV3) is a border area between ON and OFF strata in the IPL. PV0 are bistratified cells.

	Mean depth (sd)	Dendritic Field mean Area diam		Receptive Field diameter [μm]			Surround Inhibition	Transient/Sustained	Spatial Contrast Sensitivity
	%	[10^-3^mm^2^]	[μm]	BS	WS	NatS			
**PV7**	136.3 (10.0)	11 (3)	118	<125		76	Complete	Transient	Yes
**PV6**	134.0 (13.8)	42 (16)	232	291	235	200	Weak	Sustained	Yes
**PV5**	68.4 (4.0)	58 (11)	271	384	330	220	Moderate	Transient	No
**PV4**	68.8 (3.5)	24 (6)	173	178	165	96	Strong	Transient	No
**PV3**	43.3 (6.2)	11 (4)	121	<125		NA	Complete	Sustained	Yes
**PV2**	23.9 (7.2)	25 (6)	180	221	207	106	Strong	Transient	Weak negative
**PV1**	-40.8 (9.3)	53 (15)	260	467	360	156	Weak	Sustained	Yes
**PV0**	98.5 (1.8)	31 (12)	199	135		176	Complete	Transient	No
	-2.2 (2.3)	28 (18)	189		184		V.Strong	Transient	No

From 52 paraformaldehyde-fixed PV retinas (obtained from single-cell recording experiments) immunolabelled for YFP along with the nuclear marker DAPI, we estimated that 11.8% ± 5.2% (mean ± standard deviation) of all ganglion cells express YFP, assuming 40% of somata in the ganglion cell layer (GCL) are ganglion cells with a density of 8,000 ganglion cells per mm^2^ [35]. The proportions of each YFP-labelled ganglion cell type are unknown, as YFP is unlikely to be expressed in all cells of a given type. Each ganglion cell type is thought to regularly tile the retina with different levels of dendritic overlap [1, 4]. PV5 cells were often electrically coupled to other ganglion cells (S1(a) Fig), most of which were also YFP-expressing PV5 cells (not shown). PV7 cells were coupled to small cells within their dendritic fields (S1(a) Fig), which was also observed for PV5, PV4 and PV2 cells, and are likely to be narrowfield amacrine cells. Ganglion cells with larger dendritic fields (e.g. PV1, PV5) are thought to occur less frequently than cells with smaller dendritic fields. We observed a range of dendritic field areas per PV cell type, with larger PV cell types having the greatest range (S1(c) Fig). The smallest PV cell had a mean dendritic area of 0.006 mm^2^ (84 μm diameter) and stratified at 125.5 ± 4.9% depth (a PV7 cell, S1(c) Fig), and the largest PV cell had a mean area of 0.12 mm^2^ (384 μm diameter) and stratified at -45.5 ± 8.7% depth (a PV1 cell, not shown). Ganglion cells can be grouped by the combination of stratification depth and dendritic field area (S1(c) Fig). Neurons with similar stratification likely receive the same glutamatergic input from presynaptic bipolar cells, suggesting overlapping responses to visual stimulation [36].

### Characterisation of receptive field vectors–the QMI method

We used a three-stage approach to characterise the RFVs. In stage 1 we identify the RFVs using QMI and then to compare different RFVs in the stages 2 and 3 we used the MI, rather than QMI, as this provides an absolute measure of the dependence of the distributions. Details about calculating QMI, MI and the optimization are described in Methods.

1. *Number of Classes/Vectors*: The number of vectors (*K*) that the QMI technique can resolve depends on the number of classes (*M*) of the outputs: *K* = *M*– 1. We can set *M* arbitrarily, but a limit is that a vector can be resolved only if there are sufficient inputs (>50) for each output class. Here we investigate the simplest case of two class labels: spiking and non-spiking, and only one RFV.
2. *Receptive Field Radius*: To determine the relevant radius of the RFVs the Mutual Information (MI) between neuronal response classes and transformed stimulus (projections) was calculated for an expanding central circular section across the complete frame history. We first divide the dataset (input vectors and output average number of spikes) into “training” and “test” datasets, then calculate the RFV using the training dataset. The MI dependence on radius is then calculated for the “test” dataset using the previously obtained RFV. The point when the MI reaches the maximum and starts to decrease, due to overfitting, was then used to estimate the receptive field radius (*R*_MI_).
3. *Frame History (or Cell Memory)*: To determine the number of frames relevant for the RFV the MI was calculated successively for increasing number of frames, as this measure allows comparisons between different subspaces. The data were again separated into training and test sets as in 2. As an estimate of the relevant frame history we take the point where the MI reaches a maximum and starts to decrease. The frame history MI diagrams can be used to estimate the corresponding *memory* for each cell type.

**Fig 1:**
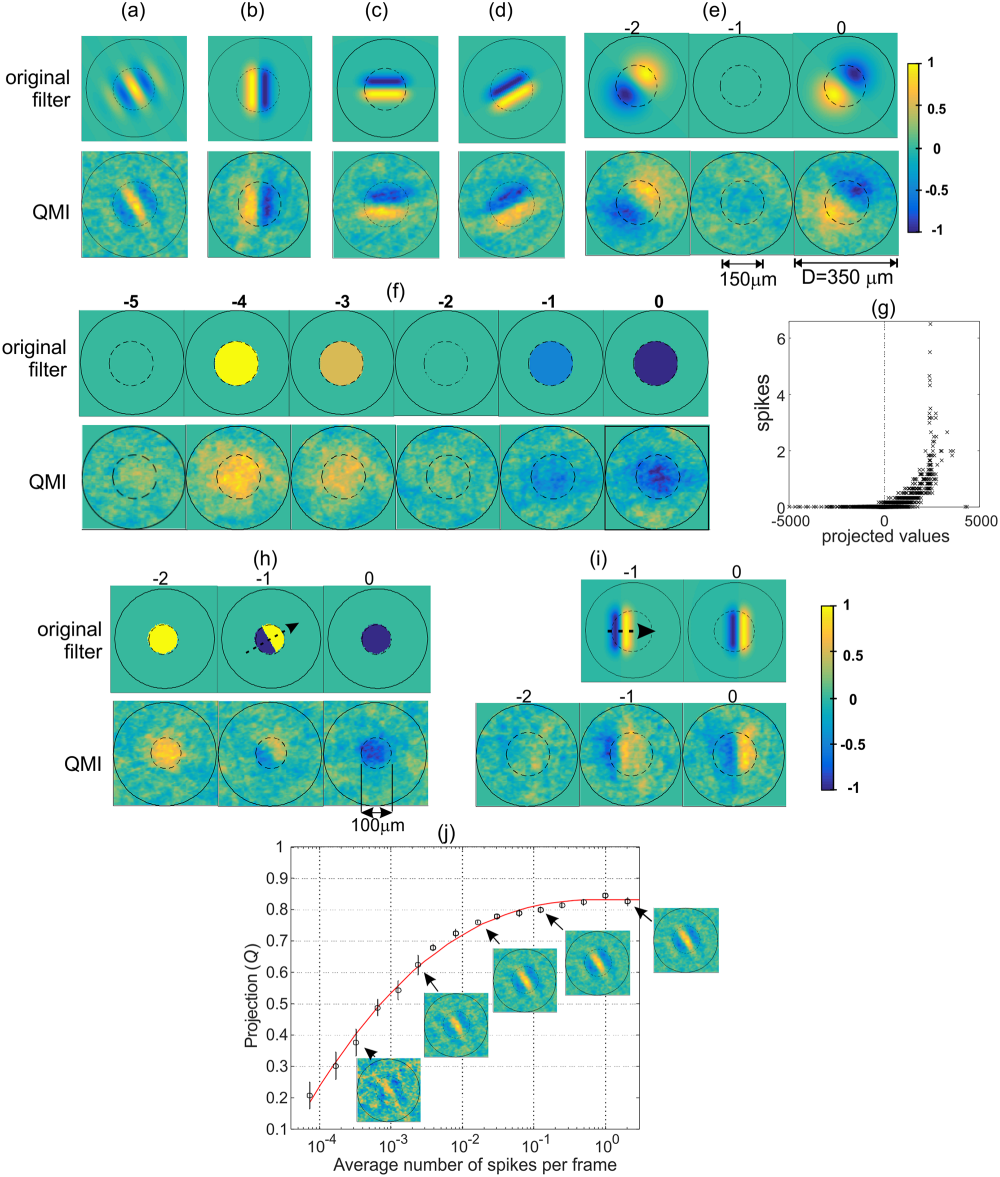
Validation of the QMI approach. Reconstruction of a model neuron RFV: the spatio-temporal filter of the model cell (original filter) and the extracted RFV in response to natural stimuli (QMI filter, shown below each original filter). Each stimulus vector (***x***) consists of *T* frames of the size 101x101 pixels, total number of stimulus frames was 3175. The nonlinearity used was half squaring (positive filter responses squared, negative set to zero). The synthetic cell response was determined by averaging the number of spikes per frame for 25 repetitions of the image sequence. The average of the total number of spikes for the complete stimuli is denoted 〈*spikes*〉. A, Gabor filter (σ_*x*_ = 60μm, γ = 1.3, λ = 94μm, θ = π/6), *T* = 1, 〈*spikes*〉 = 268, stand. dev. = 20, *Q* = 0.79. Gabor filter: $F\left(x,y\right)=\text{exp}\left[-\left(\frac{x_{\theta }^{2}}{2\sigma_{x}^{2}}+\frac{y_{\theta }^{2}}{2\sigma_{y}^{2}}\right)\right]\cdot \text{cos}\left(2\pi \frac{x_{\theta }}{\lambda }\right),\;\;\sigma_{y}=\sigma_{x}/\gamma ,\;x_{\theta }=x\;\text{cos}\theta +y\;\text{sin}\theta ,y_{\theta }=-x\;\text{sin}\theta +y\text{cos}\theta$. B, vertical edge filter (*T* = 1, length 100μm, peak-to-peak separation 45μm, 〈*spikes*〉 = 427, *Q* = 0.78). C, horizontal edge (*T* = 1, 〈*spikes*〉 = 733, *Q* = 0.77). D, arbitrary angle (*θ* = 30°, *T* = 1, 〈*spikes*〉 = 635, *Q* = 0.81). E, Filter created by subtraction of two time-varying 2D Gaussians functions (σ_*x*_ = σ_*y*_ = 60 μm, separation 37.5 μm, *T* = 3, 〈*spikes*〉 = 644). F, Center-OFF filter (150μm diameter, *T* = 6, 〈*spikes*〉 = 304). G, Average number of spikes vs. projection of input vectors onto RFV, case (f). H, “Aperture cell” filter, detecting a moving edge (*θ* = 30°, *T* = 3, 〈*spikes*〉 = 209). I, Moving bar filter (*T* = 2, length 100μm, 〈*spikes*〉 = 372)–we added one more frame in the recovered filter to show that the QMI correctly returns an “empty” frame. J, Projection (*Q*) of the recovered filter vector on the original filter (Gabor filter case (a)) vs. average number of spikes per frame. Plotted are the average values for *Q* and error bars (n = 7 repeats). The same natural scene stimulus of 3175 frames used in each case. Insets show typical recovered filters for a selection of points (the average total number of spikes is: 2, 10, 75, 468 and 6496).

In order to test and validate the use of QMI as a cost function and establish that the method works appropriately we have first demonstrated the technique using model cells with predefined spatio-temporal filters. For the input we used natural scene stimuli consisting of 3175 frames at a resolution of 101x101 pixels (S2(a) Fig and Methods). Characteristic spatial and temporal filters have been chosen for the synthetic cell model to demonstrate the ability of the QMI to recover different single frame (e.g. Gabor) and multi-frame filters (OFF-filter, direction selective, etc). The filters were correctly reconstructed (Fig 1(a)–1(i)) even for relatively small numbers of spikes (typically several hundred). As a measure of the accuracy of the recovered filter we used the projection (*Q*) of the recovered filter (normalised value $\hat{w}$) on the original filter $\hat{(e}):\;Q\;=\;\hat{e}∙\hat{w}$. The QMI technique was robust Fig 1(j), yielding an accurate filter recovery for a relatively short stimuli sequence (3175 frames) and an average number of spikes per frame above 0.01. One limitation on the maximal value that can be achieved for *Q* (≈ 0.83 in the case shown in Fig 1(j)) is the quality of the stimulation images/movies, i.e. its ability to provide sufficient relevant variability needed to detect the filter. Once this stimuli data reach sufficient quality, a relatively small number of spikes (a few hundred) are sufficient for a good quality filter recovery using the QMI technique.

**Fig 2:**
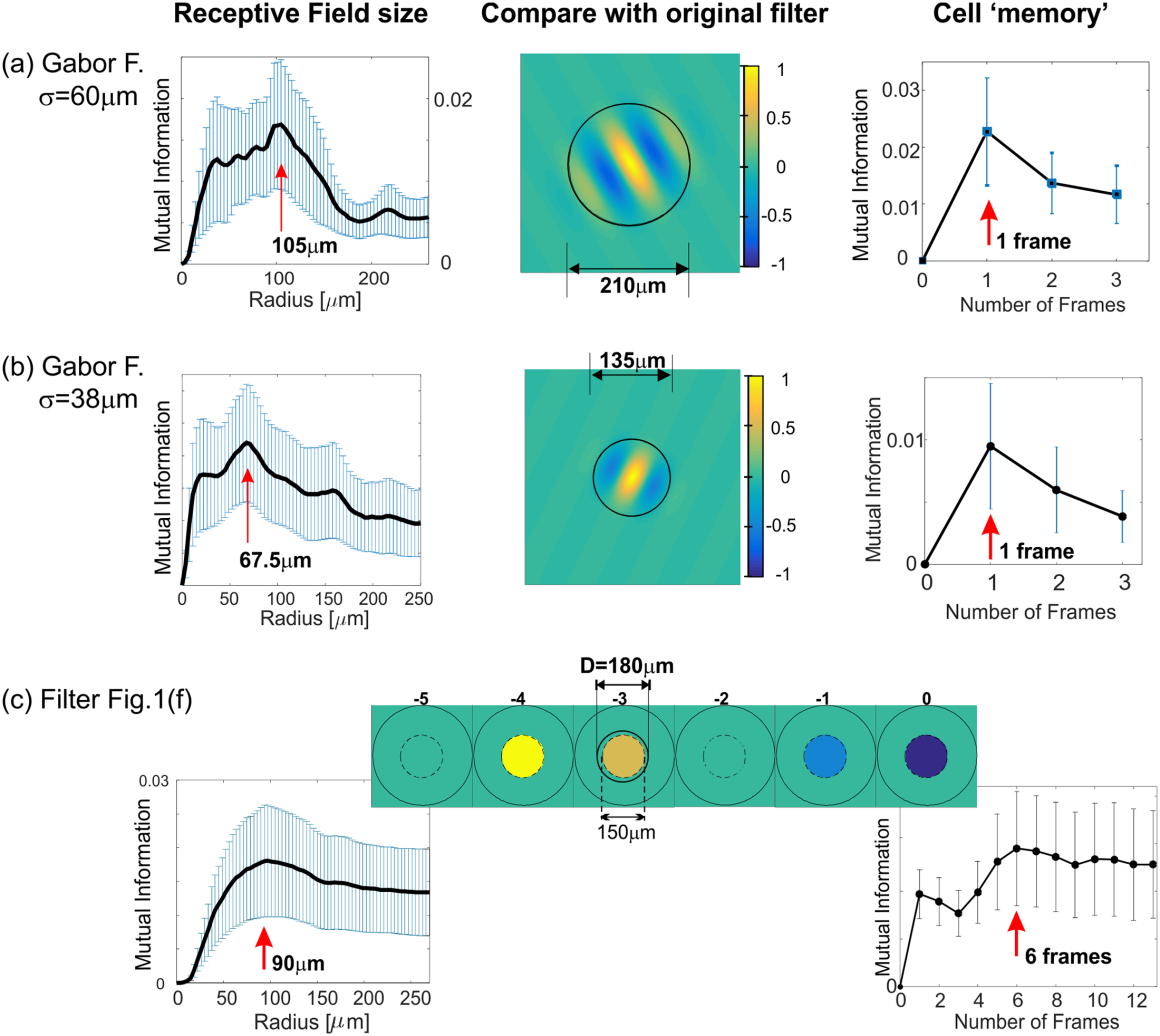
Validation of using MI to estimate the Receptive Field Radius and the Cell Memory. Left column, MI contained within an increasing radius. The decrease in MI indicates overfitting (see Results). The vertical red arrow indicates the identified radius before the onset of the overfitting artifacts (*R*_MI_). Middle column, the original filter with a circle of the radius *R*_MI_. Right, MI vs. number of frames that the RFV contains. The relevant receptive field history (or the Cell Memory) was estimated as for the radius and marked with a red arrow. (a) Gabor filter, same as in Fig 1(a), average number of spikes 650. (b) Gabor filter: σ_*x*_ = 38 μm, γ = 1.3, λ = 94 μm, θ = −π/7, 〈*spikes*〉 = 642. (c) Same filter as in Fig 1(f).

**Fig 3:**
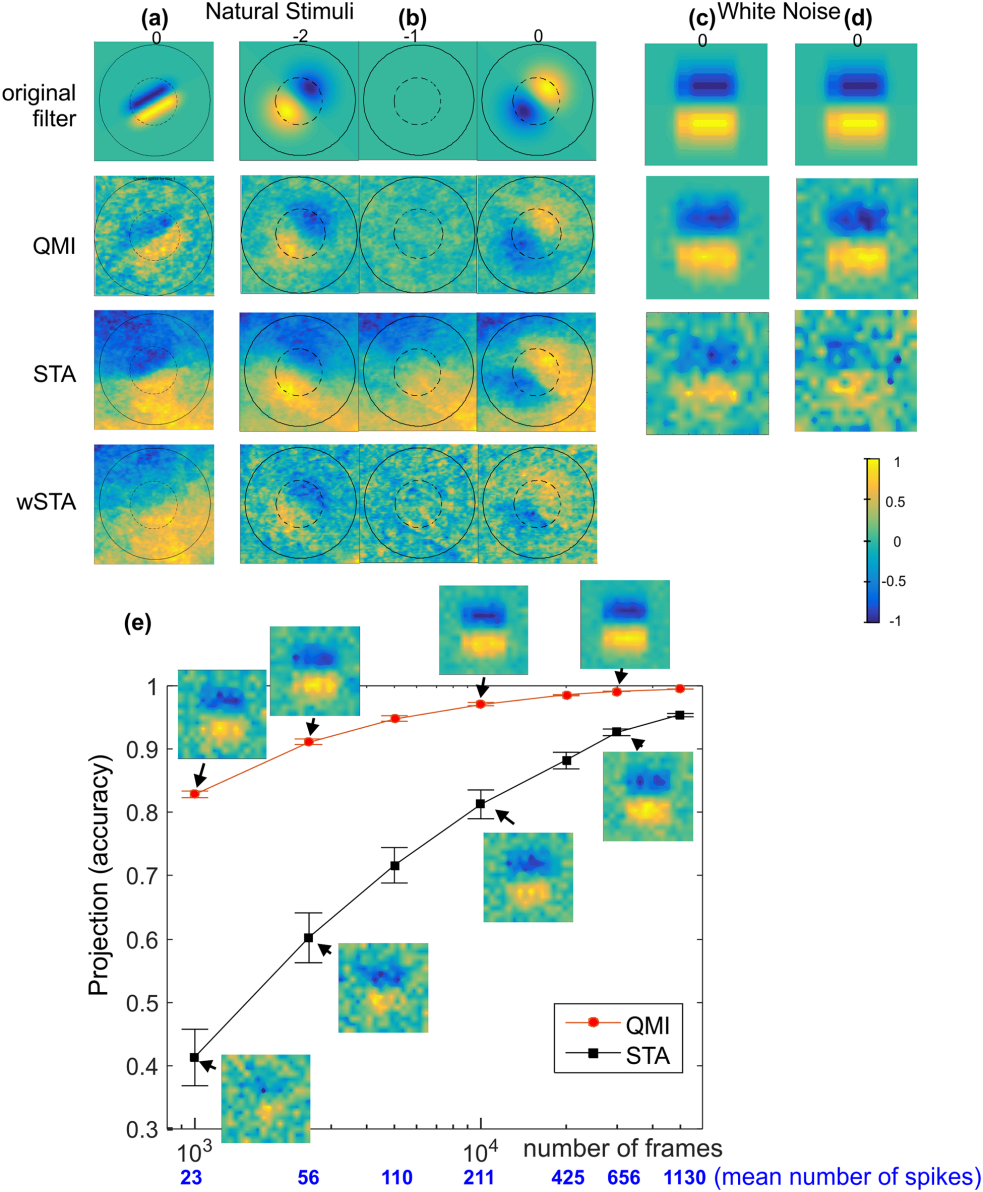
QMI compared with STA technique. The original and recovered filters using the QMI, STA and wSTA techniques. Reconstruction in the case of: natural stimulus (101x101 pixel frames) (a) filter from Fig 1(d), (b) filter from Fig 1(e), and white noise with (c) uniform and (d) Gaussian distributions, 16x16 pixel frame resolution input. (e) Direct comparison in accuracy between QMI and STA using the RFV projection measure (*Q*) vs. number of frames (and spikes) in the stimuli. Insets show the recovered filters for a selection of points. It is known that if the input signals are not white then the STA is a broadened version of the original filter; STA panels in (a) and (b). The attempt to remove correlations by multiplying the STA recovered filter by the inverse of the *a priory* covariance matrix (wSTA) doesn’t help much because the natural scene input is non-Gaussian, as discussed in detail in [25].

QMI in combination with MI can be used to correctly estimate the Receptive Field Radius and Cell Memory (Fig 2) To further test the QMI technique we used uncorrelated inputs in space and time, specifically white noise with uniform and Gaussian distributions (Fig 3(c) and 3(d)), demonstrating that the technique is equally able to use uncorrelated inputs (white noise) as well as highly correlated inputs (natural stimuli). Finally, comparisons were made with conventional methods of characterising neural feature selectivity, namely Spike Triggered Average (STA) and Spike Triggered Covariance (STC) techniques (Fig 3). QMI is very robust for reduced stimulus length and significantly outperforms STA (Fig 3(e)) as well as the STC (not shown).

### PV cells’ visual responses to natural scenes

**Fig 4:**
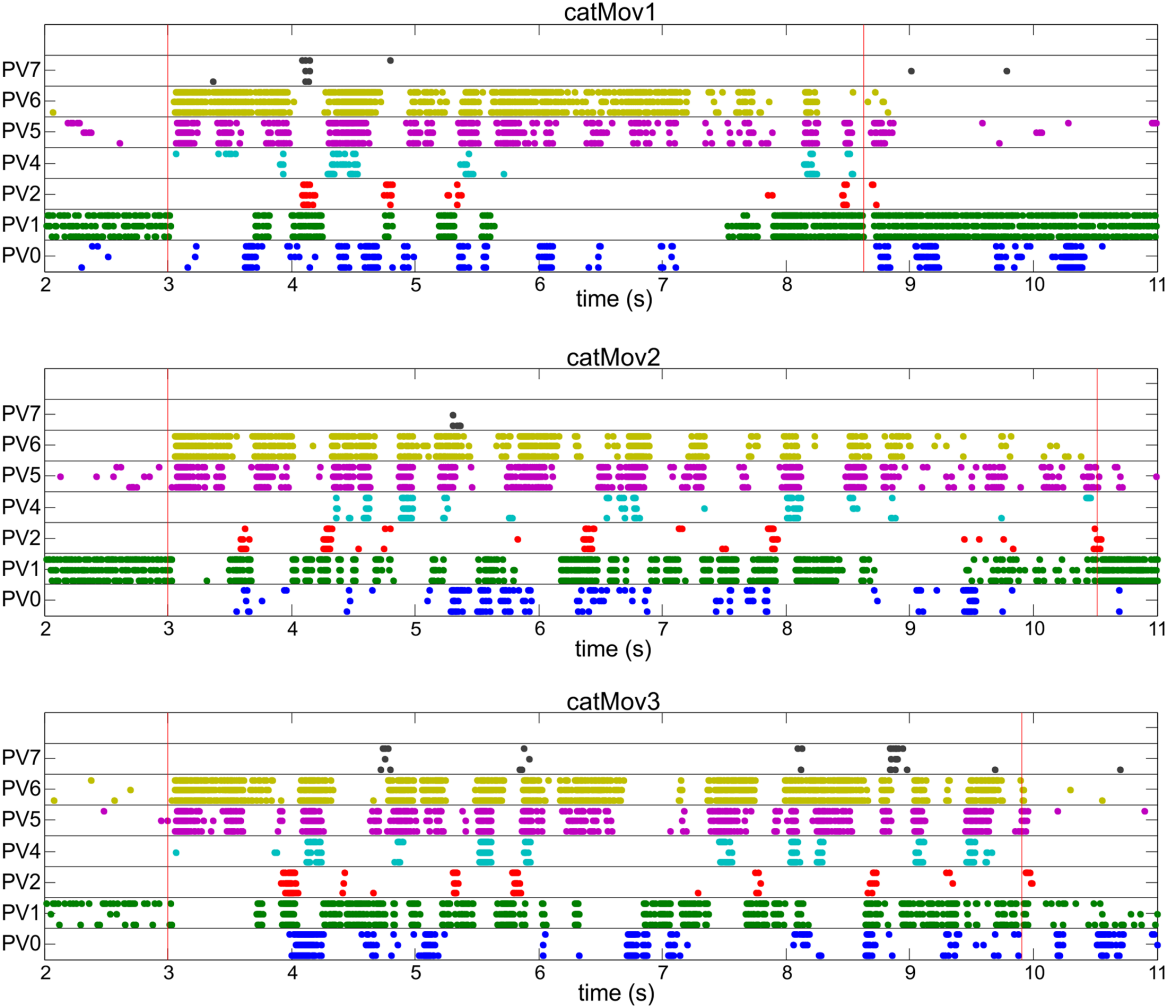
Response of the eight PV-retina cell types (PV0-PV7) to three natural scene movies (labelled catMov1, catMov2 and catMov3). For each cell type a different colour is used and three individual recordings are shown in a line. Each point represents a spike and red vertical lines represent the start (at 3 s) and the end of the movies. Before and after the stimuli the retinas were exposed to uniform gray illumination. The stimulus movies were centred around each individual cell being recorded from, i.e. every single recorded cell had approximately identical input. Additional raster plots are shown in S4 and S5 Figs.

The natural scene stimuli consisted of sequences of frames projected onto the wholemount retina typically at 25 frames per second (S2(a) Fig and Methods). Raster plots of the PV retina responses to natural scene movies revealed cell-type-specific spiking activity (Fig 4, S3 and S4 Figs), with cell types occupying the same stratum (e.g. PV4 and PV5) discharging at the same time during a subset of similar events in the natural scene. Visual stimuli were always aligned to the centre of the recorded cell’s receptive field, so that the spiking responses to natural scene movies could be compared for each cell type. In our framework, the RGC neural circuit behaviour is captured in the RFVs: a single, or set, of spatio-temporal vectors embedded in the stimulus space. The RFVs calculated here represent the particular visual stimulus input that maximally separate different neural circuit responses [37]. The stimulus space is formed by the light intensity of the individual pixels that make up the frames of the movie presented. Detailed exploration of such a large space is experimentally unfeasible, but identifying the RFVs is vital in identifying the functional behaviour of the RGC. The optimization was run for ten sequential frames history and full spatial size (320x240 pixels).

**Fig 5:**
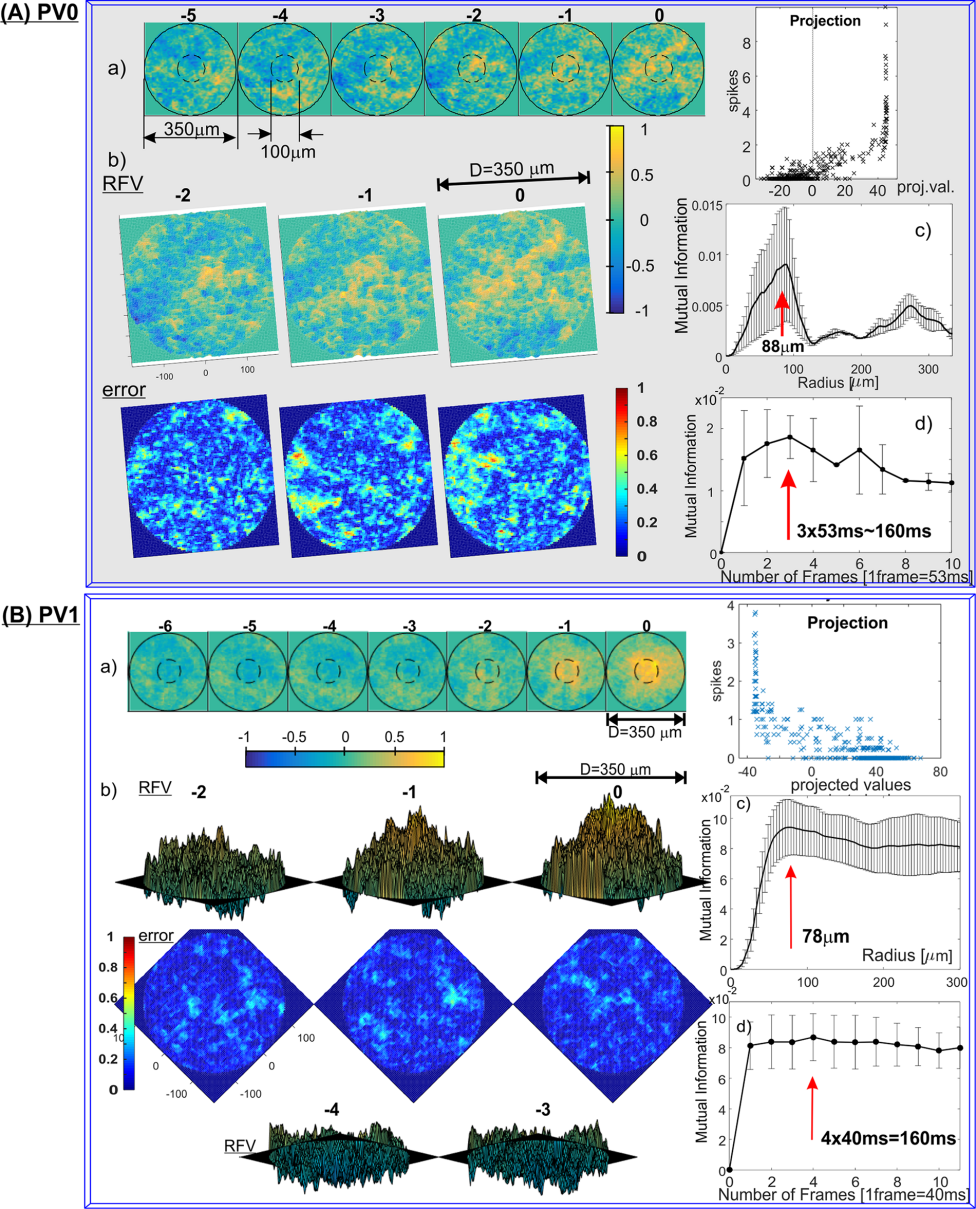
Receptive Field Vectors and estimates of the RFV radius and cell’s memory for: (A) PV0 and (B) PV1 cell types (results for representative cells shown). a) Single RFV that maximally separate spiking from non-spiking inputs. Brighter responses represent higher (illumination) values; color bar shown below (colourmap is Matlab parula, range is [–1,1]. Two circles (diameters 350μm and 100μm), assist in the estimation of the size of the structures. Right, average number of spikes generated vs. projections of the input vectors onto the RFV. b) 3D plots of the RFVs and standard error, estimated as described in Methods. PV0: azimuth = 10°, elevation = 80°, PV1: azimuth = -45°, elevation = 10° (frames: 0, -1, -2) and elevation = -10° (frames: -3 and -4). Standard error plots: same azimuth, elevation = 90°, note different colourmap (Matlab jet, range is [0,1]). c) MI contained within an increasing radius across the entire RFV. The decrease in the MI indicates overfitting (see text for explanation). The vertical red line represents the identified radius before the onset of the overfitting artifacts (*R*_MI_). d) MI vs. number of frames that the RFV contains. The relevant receptive field history (or the Cell Memory) was estimated as in c) and marked with a red arrow.

**Fig 6:**
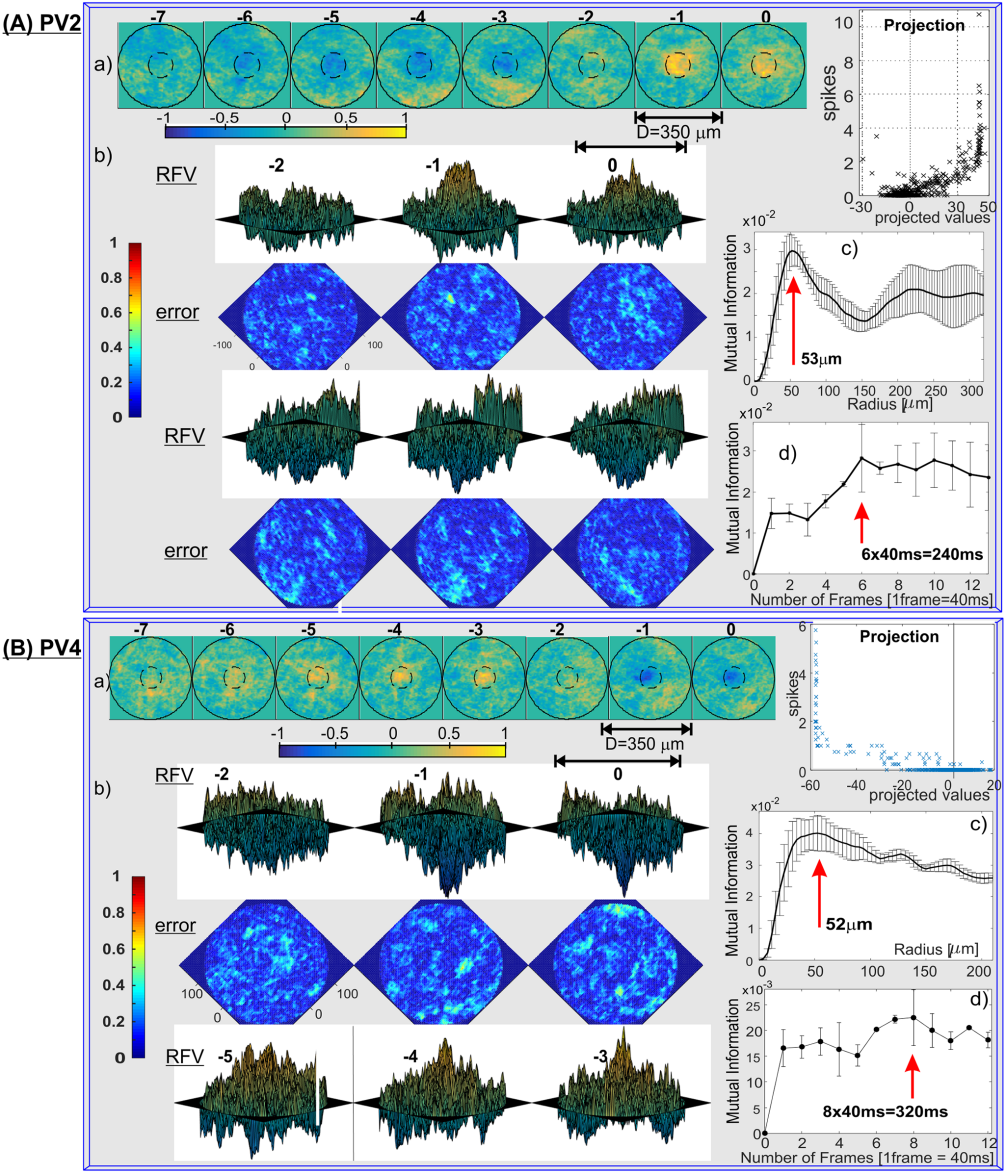
RFVs for: (A) PV2 and (B) PV4 cell classes. a) 2D RFVs and average number of spikes vs. projections of the input vectors, b) 3D RFVs with 2D error plots c) receptive field radius and d) cell’s memory estimates. Azimuth = 45°, elevation = 10° (Frame 0, -1, -2 for PV2, and -3, -4, -5 for PV4), and -10° for the rest of the frames.

**Fig 7:**
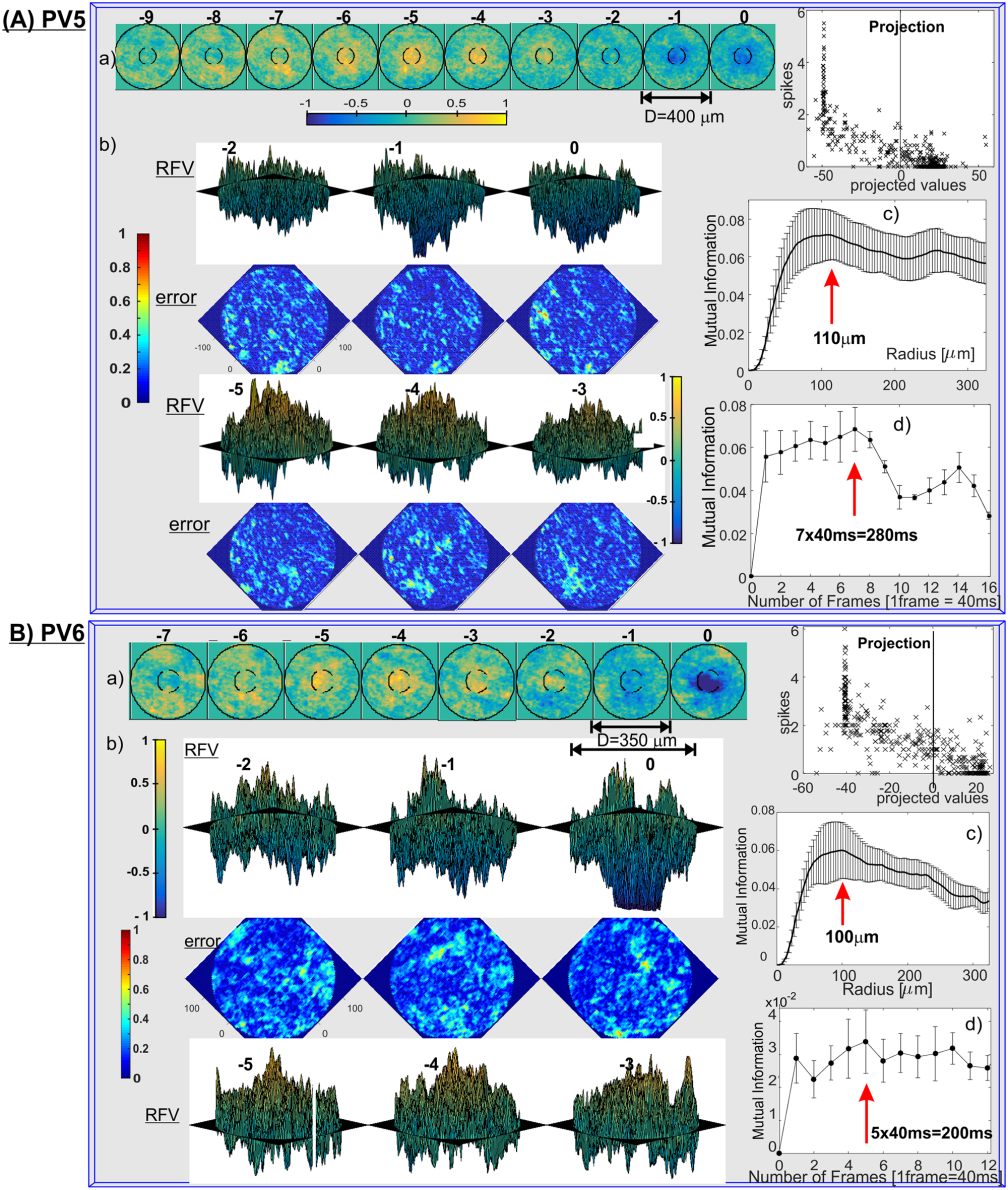
RFVs for: (A) PV5 and (B) PV6 cell classes. a) 2D and b) 3D RFVs with error bars and average number of spikes vs. projections of the input vectors, c) receptive field radius and d) cell’s memory estimates for cell types PV4 and PV5. Azimuth = 45°, elevation = –10° (Frame 0, -1, -2), and 10° for the rest of the frames.

**Fig 8:**
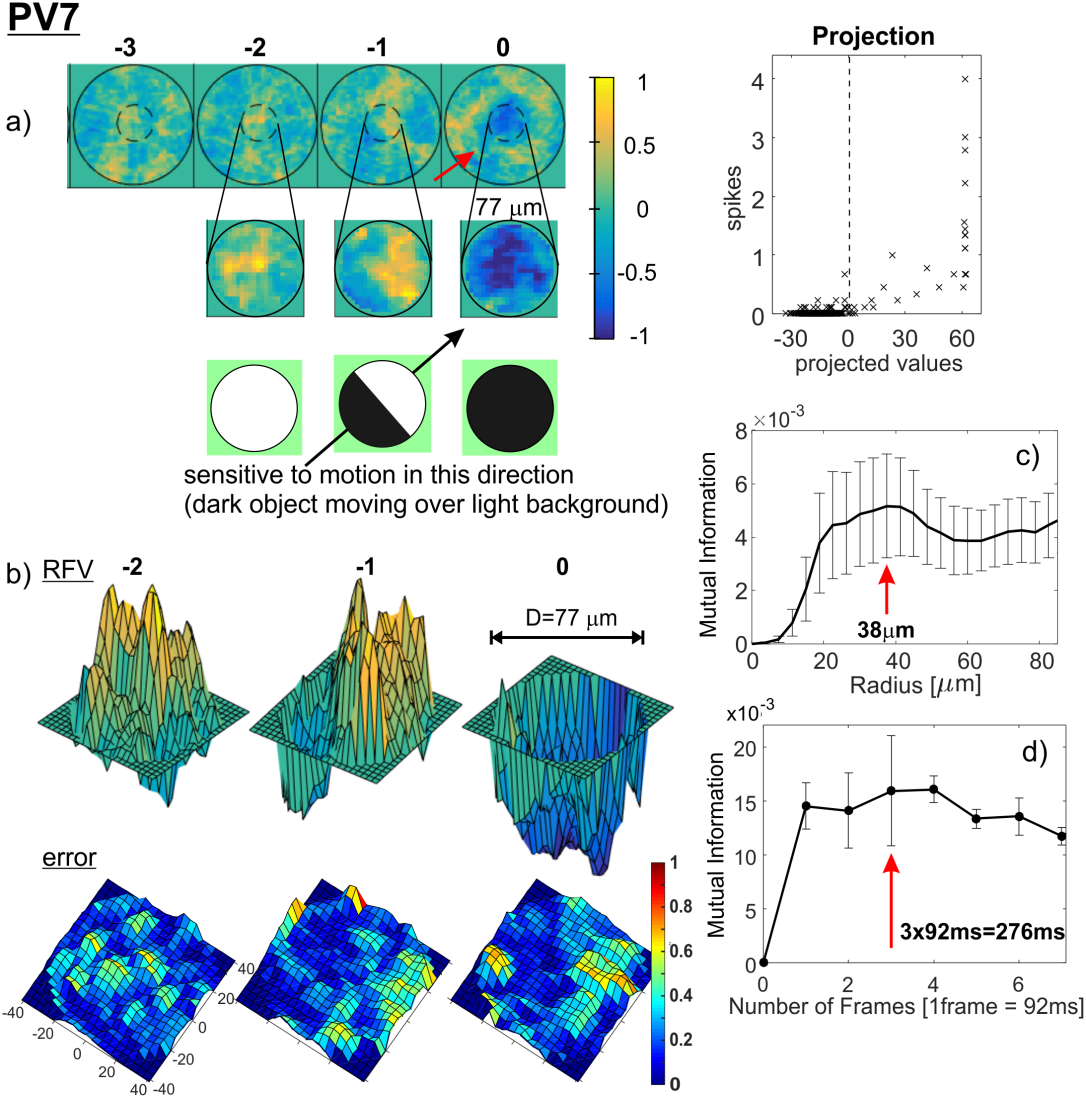
RFV, receptive field radius and cell’s memory estimates for cell type PV7. Outer circle diameter is 250μm, the inner circle diameter 75μm. Panel a), bottom shows interpretation of the biological meaning of the discerned RFV. 3D RFV: azimuth = 37°, elevation = 20°, error: elevation: 80°. Average standard error: 0.18.

The single RFVs for all PV-cell types (except PV3, whose firing patterns were too sparse for RFV calculations) are shown in Fig 5(a) (PV0 and PV1), Fig 6(a) (PV2, PV4), Fig 7(a) (PV5, PV6) and Fig 8(a) (PV7). 3D plots of the corresponding RFV are shown in panels (b), together with the estimated standard errors (see Methods for a detailed explanation). The estimated Receptive Field Radius and Frame History are shown in the same figures in panels (c) and (d) respectively.

The spatio-temporal structures of the RFVs reveal characteristic patterns for each cell type. PV0 appears to be selective to a white bar on a dark background (Fig 5A(a)). This sensitivity to the motion of a bright bar over a gray background is much clearer from S5(a) Fig. The RFV of PV1 cells appears to have a very simple structure: it begins with weak and diffuse signals then increases to a strong bright signal in a pseudo-circular region at the centre of the receptive field in frames -1 and 0 (~0–80ms). Lack of the negative phase during frames -5 to -2 could be because the cell is an ON-sustained type. PV2 cells are sensitive to brightening of the central part showing a typical dampened sine wave intensity shape with maximum intensity at frame -1 (40–80ms) and minimum at frame -4 (120–160ms). PV4 and PV5 show similar patterns (typical for transient cells): first brightening in the centre, followed by a rapid darkening. This darkening decreases in the last frame for PV4, but for PV5 the last frame has the widest and darkest middle spot. This corresponds to the previously determined function of the PV5 cell as being approach-sensitive and hence strongly responding to an enlarging black disk [8]. The PV6 cells when operating as switch have very short memory (one or two frames) and respond to a darkening area within the centre of its receptive field. This central region is surrounded by diffuse areas that are weakly sensitive to bright signals in the visual stimuli (standard “centre-surround” response). PV7 cells show sensitivity when an edge of a dark object moves on a bright background. The last frame of the PV7 RFV Fig 8(a) shows a very similar two-dimensional spatial profile to the result in Fig 2(e) in ref. [38], which was obtained by using randomly flickering bars. The structure of the receptive field consists of a dark circle in the middle surrounded by a neutral ring and then a light area. There is a gap for the preferred (incoming) direction for dark spots, indicated by an arrow.

In addition to single cell examples (Figs 5–8), we have investigated the RFV similarities and variability across the population to present variability between multiple cells of the same type, such as the RFVs for seven PV5 cells (S6 Fig). Details about the number of recordings, number of frames with zero and non-zero spikes and total number of spikes are included in the table shown in S6 Fig. The RFVs for all cells are similar, and show a characteristic OFF stimulus pattern, where the frame zero has the strongest light-OFF central pattern. Cells 2–7 have approximately the same cell memory (7–8 frames), because they correspond to movies presented at 25 frames per second, whereas the cell 1 had 11 frames per second and consequently a cell memory of ~4 frames.

The plots on the right-hand side of the panel (a) in Figs 5–8 demonstrate how the input stimuli are well separated into non-spiking and spiking in respect to projections of the input vectors on the RFV and the corresponding average number of spikes generated. However, by using only one RFV it is possible to separate only two classes of outputs (which we have selected to be non-spiking and spiking). This is forcing all spiking inputs into one (spiking) class and hence a large range of spike counts correspond to only a small range of projected values. Hence we extended the methodology of optimising QMI to an arbitrary number of classes *M*, and consequently *M*-1 mutually orthogonal vectors ***W*** = [***w***_1_, ***w***_2_,…,***w***_K_]. Use of a larger relevant subspace spanned by several vectors achieves better separation of the input vectors on the basis of their projections on ***W*** and each cell’s response. For example S5(b)–S5(d) Fig shows two vectors (***w***_1_ and ***w***_2_) for a PV5 cell, and three classes of outputs. The values of the projections are important when comparing different clusters. A projected value of 0 means that the cluster is not affected by the specific vector ***w***_i_, whereas opposite signs means that clusters have inverse dependencies on ***w***_i_, and different values of the same sign mean clusters are affected by differing amounts by the ***w***_i_. For example in S5(d) Fig the cluster C2 (representing more than one spike on average per frame time) is positively correlated with ***w***_2_. This tells us that ***w***_2_ represents an excitatory feature that the cell is most strongly tuned to and that its inverse is probably inhibitory as it has a strong negative correlation with class C0 (zero spikes).

### Additional quantification of PV cell responses to simple visual input

Some results for the spot stimulus have been already presented in ref. [7]. We have conducted a detailed analysis of the experimental recordings and a summary of those results are reported here as additional information which helps in identifying the biological role of the RGC types. Our quantitative approach was based on strict biophysical parameters, including the receptive field size, surround inhibition ratio, transient/sustained firing (also dependent on the light intensity), bimodal sensitivity to static spatial contrast, and response latency (Table 1 and S1 Table).

The spot stimulus consists of sets of black spots (BS) and white spots (WS), of different diameters, on a gray background (see Methods, also see Fig 2 in ref. [7]). Light-evoked spiking activity was measured from 286 PV cells, and each cell was classified as one of the 8 PV cell types based on either the *post hoc* analysis of the dendritic tree (for n = 182 cells; S1(a) Fig, Table 1) or directly from *ex vivo* two-photon z-stacks of the targeted cell (n = 104 cells). The general behaviour of the PV RGC types corresponds to their dendritic terminal stratification in the IPL where they receive the glutamatergic inputs from ON-BCs or OFF-BCs. Inhibition to the ganglion cell excitatory circuit, via amacrine cells, is dependent on the glutamatergic input to those amacrine cells, which can arise from many different bipolar cell types. Precise timing of these excitatory and inhibitory inputs, as revealed by whole-cell voltage clamp recordings, is critical for the functioning of these retinal circuits and will be explored elsewhere.

PV0 cells are ON/OFF transient cells (respond to both ON and OFF signals). They are bistratified and highly direction selective compared to all other PV cells (explained below). They have strong surround inhibition and small spatial contrast sensitivity [39], S1 Table. PV0 comprises one or several of the direction selective ON-OFF cell types (as opposed to ON-type direction selective [40] and OFF-type direction selective [38]), previously identified in mouse and rabbit retina [41, 42] and its circuitry well-characterised [43, 44].

PV1 are ON sustained cells. These cells have a large dendritic tree and they resemble the previously described mammalian ON-alpha ganglion cell type [45–47]. They show sensitivity to spatial contrast (the sustained component intensity depends on the spatial contrast), and have weak to moderate Surround-Inhibition-Ratio (SIR), S1 Table. The SIR for PV1 cells depends on the absolute level of illumination for the transition between scotopic and photopic illumination, due to activation of a switch-like component in the neural circuit of the PV1 cells [7].

PV2 are ON transient cells. This cell type exhibits a moderate transient light-ON response with strong but not complete surround inhibition. PV2 cells have a medium size dendritic field. PV2 cells have a pure ON motif, i.e. no response occurs at light OFF.

PV3 cells show sustained responses, and their dendrites are located approximately at the middle ‘logical’ part of the IPL (~44%, sd 6.2%) where they can receive both OFF and ON pathway inputs. PV-3 cells have complete surround inhibition. They have the longest latency time of all PV cells (S1 Table), which could be due to fast transient inhibition at the onset of light ON and OFF which blocks spiking in the first ~200–300ms, e.g. rabbit local edge detector [4]. Therefore, temporal contrast information may not be relevant for their biological function, but how about spatial contrast? Their dendritic tree field is small (D = 121 μm, n = 10) and since 1° of visual angle corresponds to 31 μm on the retina [48] that means that PV3 responds optimally to only ~4°. This suggests that they could be sensitive to high spatial frequencies and help in detection of small objects. Sensitivity to spatial contrast is significant (SCI mean = 0.37, sd = 0.5). This cell type is similar to ‘local edge detectors’ in rabbits [4, 49]. An RGC cell type with very similar morphological properties, but with transient response, were described as a selective feature detector in mouse retina [50], which is able to detect small moving objects of the size of the receptive field size of bipolar cells, but only if the background is featureless or stationary.

PV4 and PV5 are OFF transient cell types. They are in the same OFF stratum in IPL around the middle of the OFF regions (~69%, Table 1), but PV4 dendritic field area is much smaller. PV5 is the fastest cell of all PV-retina ganglion cells (peak response latency: mean = 57 ms, sd = 19 ms, n = 12), with the highest firing rate peak (median 500 Hz, S1 Table). PV4 shows nearly half of the PV5’s spiking rate and about 25% less total number of spikes. The two cell types also differ in surround inhibition, which is moderate for PV5, but strong for PV4. An equivalent cell type to PV4 might be OFF ‘parasol’ cell types in rabbit retina [51] and possibly those described in primates [52]. PV5 cells have been investigated in detail in [8].

PV6 are OFF-sustained spiking cells. PV6 cells have a large dendritic field (mean diameter 232 μm) as well as the estimated receptive field, only slightly smaller than PV5 or PV1, S1 Table. The surround inhibition is similar to PV5, relatively small (SIR mean ~0.3), indicating that the inclusion of the surround does not create a significant inhibitory effect. PV6 and PV1 are somewhat mirrors of the OFF and ON pathways. They both produce sustained response and although purely responsive to OFF (ON) stimulus, they are both sensitive to the complementary input, i.e. ON (OFF), in the sense that if they are already spiking this input will produce inhibitory current to silence them, e.g. see [8] for current plots. PV6 cells show strong sensitivity to spatial contrast. Similar cell types have been previously described as OFF-delta in cat [45], OFF-alpha in mouse [47] and OFF-delta in rabbit retina [4].

PV7 are OFF-transient cells. Their dendritic trees stratify high in the OFF region of the IPL. They have a small dendritic field (118 μm mean diameter, sd = 18 μm, n = 20) and an asymmetric dendritic tree with respect to the soma (S1(a) Fig). All PV7 cells in each PV retina were found to point in the same direction, hence they are expected to convey some ‘directional’ information. PV7 cells have a rapid and complete surround inhibition. PV7 can be sensitive to spatial contrast, although a transient-response type (see Discussion). A similar cell type has been described that responds to upward motion and were named J-RGCs because the cells express the JAM-B gene [38]. J-RGCs and PV7 are both OFF direction selective cells and have very similar dendritic trees, with the additional observation that the neurobiotin labelling of PV7 cells reveals electrically coupled cells within the dendritic field (S1(a) Fig).

## Discussion

We have demonstrated that from a subpopulation of genetically-identified mouse retinal ganglion cells [7, 8] their responses to simple visual stimulation can be quantified to enable comparison of different physiological metrics between distinct cell types. It is fortunate that the kinds of cells genetically-labelled in the PV retina consist of diverse types of ganglion cells, with their dendrites stratifying at different levels spanning the entire IPL. The broad range of cell types may have facilitated their quantification, in contrast to largely unbiased surveys that inevitably sample some cell types that are similar to each other [46, 53–57]. Targeting neurons immunoreactive to calcium-binding proteins also reveals subpopulations of ganglion cells [58, 59] but lacks information on their visual responses. Transgenic mouse lines that express fluorescent reporters in one or two ganglion cell types are useful for characterising visual responses and their projections (e.g. [38, 50, 60]) yet directly comparing them to other types requires separate mouse lines and/or viral labelling strategies [61]. The PV retina is thus advantageous in being able to target up to 8 different cell types in the same experiment. We can assume that each of the 8 PV cell types independently tile the retina, since 7 of these YFP-expressing ganglion cells (with the exception of PV2) match previously defined ganglion cell types with known tiling properties. This implies that each PV cell type acts as a parallel visual channel in the retina, encoding different aspects of the visual scene [1, 4, 62].

During natural scenes, individually recorded PV cells showed a wide variety of response strengths to the same visual input (total of 502 frames and 502 input vectors), yet the RFVs within a given cell type were remarkably similar, e.g. see S6 Fig. In some cases the spatial structure of some RFV frames may reflect the underlying synaptic strength in the IPL [63], meaning that the RFVs may capture fine scale heterogeneities in spatial sampling of ganglion cells. However, we note here that lack of smoothness in the obtained RFVs is mostly due to limited data, as demonstrated for the synthetic cells in Figs 1 and 3.

Of the three PV cell types with the largest somata and dendritic fields (PV1, PV5, and PV6 "alpha-like" ganglion cells), PV1 cells have the largest receptive fields, integrating a wider visual angle than other types, which may suggest that this type of RGC is not used for some small local changes, but rather more large-scale features in the scene. However, these cells usually take input from a few hundred bipolar cells (e.g. 200–300 for PV5, each bipolar cell has a receptive field of approximately 30μm diameter and the RGCs receptive filed is 200–300 μm) and this small scale subunit structures of bipolar and amacrine cells can interact locally within the receptive filed of the large alpha cell. So in some cases small local changes matter, for example creating frequency doubling response for an input consisting of grating of particular spatial scale [63] or a change in spatial encoding depending on the light intensity [64]. However a question remains how relevant these features are for the biological role of the cells. PV1 cells can be fast in responding, but they show sensitivity to static spatial contrast and produce a sustained response. It is thus possible that they are optimised for detecting fast movement of light objects on dark backgrounds, so if a white object is fast moving the inhibition is too slow to block excitation. Furthermore, PV1 could serve the purpose of detecting a bright region in the visual field and specifically identifying the point of maximum brightness (when scanning the horizon the persistent component will gradually increase as it becomes brighter and then there is a transient spike when the light source is centred in the visual field). This feature could help finding holes leading out from dark environment (a so-called “hole-in-the-wall” detector). Similarly, this cell type could be necessary to protect the eye from sudden increases in brightness that would register as sharp transient peaks and may affect the regulation of pupil dilation [65, 66]. It is known that rodless/coneless mice still retain pupillary light reflex, and that comes from intrinsically photosensitive retinal ganglion cells [67]. This property is mediated by the photopigment melanopsin [68], which is expressed in some retinal ganglion cells including "ON alpha" cells which may be PV1 cells.

From the transient/sustained nature of the response of PV6 cells, it would appear that both the two-frame and six frame RFVs capture some aspects of the cell's behaviour. Using just two frames leads to a continuous sustained response to BS stimulus (Fig 7, Table 1 and S1 Table). Using the six-frame vector captures the transient part of the PV6 cell behaviour. This behaviour is also consistent with our hypothesis that PV6 acts as a switch: for light OFF it is "switched on" and continuously fires until there is a light ON input which will silence it.

PV5 cells show strong transient excitation but no active inhibition triggered by the light OFF signal and conversely there is a very strong inhibition for an ON event, but no excitation at all. This leads to the conclusion that if two areas of a similar size within the receptive field of a PV5 cell are simultaneously experiencing OFF and ON inputs they will create opposite synaptic inputs to the PV5 RGC and hence it will not spike. This is a typical situation after a dark object enters the receptive field and moves sideways. However, if the OFF signal is larger, then the cell will spike and this will intensify if the OFF area is increasing. This will happen if a dark object (on bright background) is approaching the animal. This cell type was first identified and the retinal circuitry explained by ref [8] and named “approach sensitive” RGCs. The MI and RFV results corroborate this conclusion, showing the correct duration of the cell's memory (~280ms) and the average receptive field size (~220μm), as in ref. [8]. The RFV starts with an expanding white spot, which then shrinks to zero and then appears an expanding black spot which darkens in intensity and ends with the maximum diameter for the last frame.

The PV4 cell RFV (Fig 6B) indicates that this cell is sensitive to a bright spot that switches into a dark spot before remaining constant. This behaviour can also be observed from the simple spot stimulation. The cells respond only to small, centred, OFF spots, but if they become larger than 250μm in diameter there is no response from the cell whatsoever. So the cell will not respond if the whole scene darkens (i.e. will not act as a 'dimming detector'). In some respects (e.g. RFVs) PV4 cells are similar to PV5 (the approach sensitive cell) but they have a smaller receptive field and they are much slower in response to the BS onset. They may also receive the same type of OFF bipolar cell input as they costratify (cyan and magenta clusters in S1(c) Fig, respectively) and have overlapping responses to some features of the natural scene stimuli (Fig 1).

PV2 cells show sparse spiking both for the spot and natural scene stimuli, indicating that it is highly selective. Firing patterns for natural scene stimuli with a lower frame rate (11fps, ~90ms frame time) are similar to 25fps (not shown). This could mean that PV2 is mostly sensitive to some contrast change and the speed of change is less important. The RFVs all show similar elongated patterns of excitation: an initial dark stripe that becomes brighter. This may be important for locating the horizon during head motion.

The temporal contrast change due to BS and WS stimuli does not provide relevant information for the biological function of PV3 cells, but this cell type is sensitive to small spatial contrast changes. The small receptive field of diameter D~120μm corresponds to a small visual angle of α≈4°. While a mouse has no fovea, the PV3 cells seem to be associated with resolving small details in the visual field. Since inhibition is extremely fast it will suppress all fast changing inputs so it is largely ignoring temporal information about the visual scene. Any input which is shorter than 200–250ms will be suppressed allowing the cell to function as a low-pass filter in time but high-pass in space (due to its small receptive field).

PV7 could have a function in the domain of direction selectivity, due to its asymmetric dendritic tree and transient response. Our analysis here and results shown in Fig 8 for the RFV support this assumption that indeed this cell is direction selective in respect of dark object moving on a bright background (Fig 8a). The receptive field of this cell is small (*D*_MI_ ~ 75μm from Fig 8c) which corresponds to ~2° of visual angle. So it could detect motion of small edges. Perhaps the function is to detect motion of small objects (e.g. bugs or similar moving food). PV3 cells also have small receptive field but much slower response so their role is probably more related with the slowly changing or static spatial contrast.

The bistratified PV0 cells receive two independent inputs: one from the ON pathway and one from the OFF pathway. Hence the inputs cannot interact on the dendritic tree, but only in the soma. This is in contrast with PV3 cells which also receive input from both ON and OFF pathways, but on the same strata hence they can directly interact. PV0 cells are highly direction selective cells what is expected from a bistratified cell. PV0 has a very prominent spatio-temporal structure in its RFV, shown in S5(a) Fig: a bright bar moving across the middle of the receptive field. It is interesting that the maximum response of this cell is when the bar first moves in the opposite to preferred direction (non-spiking or “null-direction”), stops and then moves in the preferred direction. Analysis of the inhibition and excitation interaction during the presentation of a 200μm diameter white spot moving at the speed of 900μm/s (results not shown) shows that the excitation changes faster than inhibition for the preferred direction. However, for the null-direction inhibition is faster and blocks the onset of excitatory input. So the role of inhibition is to block the cell’s response to irrelevant direction.

The RFVs calculated in this study show significant deviation from smooth, Gaussian profiles, what is in agreement with the previous measurements of ganglion cell receptive fields [69]. Finally, an important assumption here is that correlated firing between neighbouring ganglion cells in response to natural scenes does not carry visual information [70]. Hence the firing of each ganglion cell can be evaluated independently of their neighbouring ganglion cells despite overlapping in the receptive field and if they show correlated activity, but almost all of them do not [69]. This is not surprising since the ganglion cells on the distance up to 100μm are usually ganglion cells of different type, in the part of the retinal mosaic that we investigated.

The information theoretic approach used here proved efficient in dealing with natural scene, i.e. non-Gaussian stimuli, and relatively small number of spikes. The optimization process that maximises the mutual information between the neural responses and projections of the stimulus onto low-dimensional subspace yields the receptive field vectors which define that subspace. The optimization is non-parametric, except for the Parzen-window width (σ) of the spherical *d*-dimensional Gaussian kernel functions, used to estimate the probability densities. For QMI, σ is equivalent to an interaction length that determines the effect between pairs of values in the projected space. The optimisation was initialised by using a randomly generated set of receptive field vectors. The value for σ was set to half the maximum distance between the points in the projected space S to ensure interaction between all points. Then σ was increased during the first stage of optimisation until the maximum distance between the points had stabilised. By initially expanding σ we ensure that all values affect each other. A second stage was then performed using iterative reductions of σ in order for local interactions to become stronger and so find any fine detail in the point distributions.

Regarding the value of K, in this paper we limited the investigation of this parameter to initially set it to a fixed value. The algorithm works by balancing three separate "forces", see Methods eq (8):

1. attraction within a class (*V*_INT_)
2. attraction between all points (*V*_ALL_)
3. repulsion between classes (*V*_BTW_)

These act over a range (distance between pairs of data points) that is controlled by σ (the expansion/contraction that occurs, explained above). The value of *K* actually determines the maximum number of clusters that can be separated (*K*+1). However the actual number of separated classes can be less than *K*+1 due to the small class size. Our empirical conclusion is that the minimum cluster size is approximately 15–20 points. When σ is large during the initial separation, the repulsion between the classes dominates, pushing all the classes apart. During the second step in which σ is reduced, the separate classes cannot "see" each other (as they are outside of the "forces" range) so the repulsion lessens. At this point the attraction within the classes and points starts to be more dominant and this process is driven by the larger clusters as they have more points. This means that it may be favourable to group together smaller clusters if this also groups together the larger ones.

The calculation of MI and QMI based on a limited number of observations is biased (e.g. [71]) and we have estimated that effect. The QMI optimisation process is not affected with bias because the objective is to find the transformation ***W*** such that the transformed variable *Y* maximises the function *QMI*(*C*, ***Y***), see eqs (2) and (3), not the actual value for the QMI. Hence a systematic error in QMI has no effect. However, in the case of calculating the MI as a function of frame size, *MI*_*r*_ = *MI*(*r*), or number of frames *MI*_*t*_ = *MI*(frames), bias does affect the results. For example, in the case of *MI*_*r*_ the bias decreases as *r* increases and hence the maximum MI will shift to lower values for *r* if σ is constant, see Methods for explanation. This happens because σ was optimised for large ***r***. Therefore we correct for bias by scaling up the values for the σ in proportion to the span of the values for *S* for each ***r***. This approach will not necessarily completely eliminate the bias, but will even it out and hence the bias will not affect the position of maximum for MI.

It is well known that natural scene stimulus entails considerable spatial and temporal correlations [72]. We assessed correlations within frames and between frames for our natural scene movies by using the standard autocorrelation function: (a) *C*(Δ*x*, Δ*y*), which gives the correlation (average of the product) of the intensity at two locations as a function of relative distance of these locations–see S2(b) Fig, and (b) *C*(Δ*t*) correlation of intensities of the same pixel but at two different frames–S2(c) Fig. A frequent presence of a certain shape (e.g. moving bars) might affect some RFVs. However, apart from PV0 we have not identified sensitivity to that pattern in any other cell class. In general, this is not affecting the proposed technique, and any artefacts disappear with increasing number of input frames, e.g. see Fig 3.

The dimension of the stimulus space used for the optimization process is 768,000, which is 320x240x10: number of pixels in the projected images times the number of frames assumed to be relevant. By using QMI instead of conventional MI, the optimization process becomes practically feasible for such a high-dimensional stimulus space. Since the approach allows us to optimise the RFVs far more efficiently, we can search the complete stimulus space for multiple vectors simultaneously. Another crucial advantage is that this approach works very well with a small number of recorded responses, as optimising the quadratic mutual information is equivalent to optimising the lower bound of the full mutual information, preventing over-fitting of the very small sample size [32].

## Methods

### Experimental

#### Animals and data

All primary data (responses to spot stimuli and labeled ganglion cells) used in this study were obtained from *Pvalb*^*Cre*^;*Thy*^*Stp-EYFP*^ mice. The sample of retinal ganglion cells from these mice have been described previously [7]. We restate here from [7] that: “All animal procedures were performed in accordance with standard ethical guidelines (European Communities Guidelines on the Care and Use of Laboratory Animals, 86/609/EEC). The study was approved by the Veterinary Department of the Canton of Basel-Stadt (Kantonales Veterinaramt, Postfach 264, 4025 Basel, Switzerland, document no. 2105)”.

#### Retina preparation

Retina isolation was done under normal light levels in warm Ringer’s medium. Next the retina was transferred to a recording chamber (Open Diamond Bath, Warner Instruments) and mounted with the ganglion cell layer facing up on nitrocellulose filter paper (MF-membrane, Millipore, USA), by stretching the tissue off-centre over a hole in the filter paper that was ~2/3 the diameter of the retina. The retinas were superfused with Ringer’s medium at 34.5–36°C.

#### Imaging and electrophysiology

To visualize and target fluorescently-labeled neurons in the live wholemount retina without causing significant bleaching of the photoreceptors, we used a custom two-photon microscope using a modified Nikon Eclipse E600FN microscope and a Spectra-Physics Mai Tai Ti:sapphire laser (930nm) as in ref [8]. Energy output at the retina level was 5–20 mW. The fluorescence emission was detected (~500–600 nm) by a photomultiplier tube (Hamamatsu model R3896). The retina was illuminated using a Digital Light Processing projector (V332 PLUS with lens removed, 75 Hz refresh rate) with an infrared filter before the condenser lens, and detected by a EM-CCD camera (Hamamatsu model C9100). During two-photon excitation, the fluorescence signal detected by the PMT was digitally superimposed onto the infrared image detected by the CCD camera in real time using custom LabVIEW software [8]. The spiking responses of PV cells were recorded in loose cell-attached mode (10kHz sampling rate). All visual stimuli were aligned to the centre of the ganglion cell’s receptive field by initially presenting small flashing squares in a 9 by 9 grid, then moving the centre of the stimulus to the square that induced the strongest response. Note that the centre of the receptive field is not necessarily the soma, as many dendritic trees are asymmetric, even for large neurons (S1(a) Fig). Recorded cells were subsequently labelled with neurobiotin during whole-cell patch clamp recordings (not shown). Multiple single cells were often recorded in each retina. Representative two-photon images acquired during experiments were used for identifying the correct cell/area in the paraformaldehyde-fixed tissue *post hoc*.

#### Anatomical analysis and identification of spikes

We used Mathematica (Wolfram Research) to initially sort and classify data into PV cell types and for all the clustering. The method to calculate the mean stratification of dendrites is published in [8]. To identify the spikes we used a modified form of the Wave_clus *Matlab* application for unsupervised spike detection and sorting developed in [73]. This initially filters the data and uses an optimal amplitude threshold to identify the position of spikes. Feature extraction is then performed on the detected spikes and a super-paramagnetic clustering algorithm applied to group these features into like spikes. For our purposes, the code was modified so that the data was first over-sampled using a cubic spline interpolation to better identify the position of the spikes. This data was then passed through an elliptic filter with a frequency range of (300Hz, 3kHz). It was found that the recorded spikes had a biphasic form and that using a negative threshold gave the best results for correctly identifying spikes and avoid artefacts. Feature extraction was performed using the first three principal components of the spike form and the clustering set to create as few groups as possible since we are only measuring spikes from a single source.

#### Visual Stimulus

The natural scene movies were recorded from a camera strapped to a cat’s head as it freely explored a woodland, see S2(a) Fig [9, 10]. Three separate sequences were used "catmov1, "catmov2", and "catmov3" consisting of 141, 188, and 173 frames respectively. The projected video had a 320x240 pixel resolution and covered an area of 1200 x 900μm (width x height) on the retina and, normally, each frame was displayed for 40ms corresponding to a frame rate of 25fps, but other frame rates were used occasionally.

#### Statistical Analysis

All measures of statistical difference were performed using ANOVA. All data points represent mean ± SEM. The ‘‘n” in the figures refers to the number of different cells included for retinal recordings.

### Information Theory Concepts

Input. Let us assume that the each visual spatio-temporal stimulus ***x*** is represented by a vector in a *D*—dimensional space *R*^*D*^ and that an RGC is characterised by a set of *K* vectors ***W*** = [***w***_1_, ***w***_2_,…,***w***_K_]. Now for each stimulus vector ***x***_i_, a real valued signal can be produced by projecting the stimulus onto this subspace, using the inner product: ***y***_i_ = {***x***_i_ · ***w***_1_, ***x***_i_ · ***w***_2_,…,***x***_i_ · ***w***_K_}. Hence we define a transform *g*(***x***,*W*) of the natural stimuli (***x***) with respect to *W* as *g*(***x***,*W*): {***x*** · ***w***_1_, ***x*** · ***w***_2_,…,***x*** · ***w***_K_} ≡ ***Y***, and use as a measure of the input signal. An example of ***x*** given in S8(d) Fig.

Response classes. The detected RGC spikes were initially binned according to the frame rates of the presented natural stimuli (for *N* input vectors there are *N* bins). The average spiking response during stimuli presentation is a (*N*-dimensional) vector *s*. Now we note that the average number of spikes per bin is in the range between 0 (no spikes) and more than 10. Since the number of spikes after each input characterise the strength of the cell’s circuit response to that input, the cell’s response can be separated into a set of discrete classes (*C*) defined accordingly by the number of spikes. The simplest division would be to have two classes: non-spiking and spiking.

Mutual Information. *MI*(*C*, ***Y***) between the class labels (*C*) and the transformed data (***Y***), is defined as:

$$MI\left(C,Y\right)=\sum_{c\in C}\int_{y\in Y}^{}p\left(c,y\right)\text{log}\frac{p\left(c,y\right)}{P\left(c\right)p\left(y\right)}dy$$ (1)

where *P*(*c*)is the probability of occurrence of a discrete class *c*, *p*(***y***) is the probability density of a random variable ***y*** which represents transformed input vector ***x*** and *p*(*c*, ***y***) is the joint probability density for a (*c*, ***y***) pair. MI accounts for higher-order statistics, not just for second order and it can also be used as the basis for non-linear transforms.

Quadratic Mutual Information (QMI). QMI as an alternative approach to MI was proposed by Torkkola [27] because it provides an equally rigorous cost function for optimization as MI but is far more computationally viable. Defined as:

$$\begin{array}{ll} QMI\left(C,Y\right) & =\underset{c}{\sum }\overset{}{\underset{y}{\int }}{\left(p\left(c,y\right)-P\left(c\right)p\left(y\right)\right)}^{2}dy\\ & =\underset{c}{\sum }\overset{}{\underset{y}{\int }}p{\left(c,y\right)}^{2}\;dy+\underset{c}{\sum }\overset{}{\underset{y}{\int }}P{\left(c\right)}^{2}p{\left(y\right)}^{2}\;dy-2\underset{c}{\sum }\overset{}{\underset{y}{\int }}P\left(c,y\right)P\left(c\right)p\left(y\right)\;dy\;={\text{V}}_{\text{INT}}+V_{\text{ALL}}+V_{\text{BTW}} \end{array}$$ (2)

The last three terms in Eq (2) were named ‘information potentials’ [27].

Optimization. To perform the optimization we then need to solve:

$$W={\text{argmax}}_{W}\left[\text{QMI}\left(C,Y\right)\right]\;,\;\;y_{i}=W^{T}x_{i}$$ (3)

Hence the objective is to find the transformation ***W*** such that the transformed variable *Y* maximises the mutual information between transformed inputs and class labels, for example the natural video input and cell spiking. If QMI is estimated in a differentiable form then QMI can be maximised using gradient ascent of the QMI in the input space, iteratively:

$$W_{n+1}=W_{n}+\eta \frac{\partial QMI}{\partial W}=W_{n}+\eta \overset{N}{\underset{i=1}{\sum }}\frac{\partial QMI}{\partial y_{i}}\frac{\partial y_{i}}{\partial W}=W_{n}+\eta \overset{N}{\underset{i=1}{\sum }}\frac{\partial QMI}{\partial y_{i}}x_{i}^{T},$$ (4)

Where *η* is the learning rate. While the natural gradient of the QMI can be calculated directly, to ensure orthogonality of the receptive field vectors during the optimization a Cayley transform was used in combination with a Sherman-Morrison-Woodbury inversion to allow a simple gradient descent optimization to be performed, details of this technique can be found in [74, 75].

To construct the subspace we used an orthonormal two-dimensional weight matrix ***W*** (***W***^T^***W*** = **I**) of size [*K* x (76800 x *H*)], where 76800 (= 320 x 240 pixels) is the dimension of a single frame, and *H* is the number of frames used to represent the cells temporal response (frame history). The projection of these vectors was then calculated by convolving the weight vector with the stimuli frames (input vector ***x*** example shown in S8(d) Fig) to give ***Y*** with dimensions [*K* × *N*]. The quadratic mutual information can then be calculated using eq (2).

Parzen density estimator [76]. If ***y***_*i*_, = 1,…,*N* is a set of values for the random variable ***y*** then the probability density is estimated as sum of spherical *d*-dimensional Gaussians each centred at a sample ***y***_*i*_

$$p\left(y\right)=\frac{1}{N}\sum_{i=1}^{N}G(y-y_{i},\sigma I),$$ (5)

where G is the Gaussian multivariate kernel function and σ the Parzen-window width

$$G\left(y,\Sigma \right)=\frac{1}{{\left(2\pi \right)}^{\text{d}/2}{\left|\Sigma \right|}^{1/2}}\text{exp}\left(-\frac{1}{2}y^{\text{T}}\Sigma^{-1}y\right),$$

with the key property:

$$\overset{}{\underset{y}{\int }}G(y-y_{i},\sigma_{1}I)G\left(y-y_{j},\sigma_{2}I\right)=G\left(y_{i}-y_{j},(\sigma_{1}+\sigma_{2})I\right).$$ (6)

Probability distributions. Assuming that there are *J*_c_ samples of class *c*, then the class prior probabilities are (*c*) = *J*_c_/*N*, with $\sum_{c\;=\;1}^{M}J_{c}\;=\;N.$ For the probability densities *p*(***y***) and *p*(c, ***y***) we use Parzen estimates, eq (5), with symmetric kernel of width σ (assuming the same value for both distributions):

$$p\left(y\right)=\frac{1}{N}\overset{N}{\underset{i=1}{\sum }}G\left(y-y_{i},\sigma^{2}I\right)\;,\;\;\;p\left(c,y\right)=\frac{1}{N}\overset{J_{c}}{\underset{k=1}{\sum }}G\left(y-y_{c,k},\sigma^{2}I\right)$$ (7)

where variables ***y***_*c*,*k*_(*k* = 1, …, *J*_*c*_) all correspond to the same class *c*.

‘Information potentials’. Substituting Eq (7) in eq (2), all integrals transform into sums and can be defined as “information potentials” as in [32]:

$${\text{V}}_{\text{INT}}=\frac{1}{N^{2}}\overset{M}{\underset{c=1}{\sum }}\overset{J_{c}}{\underset{i=1}{\sum }}\overset{J_{c}}{\underset{j=1}{\sum }}G(y_{ci}-y_{cj},2\sigma^{2}I)$$

$${\text{V}}_{\text{ALL}}=\frac{1}{N^{4}}\left(\overset{M}{\underset{c=1}{\sum }}J_{c}^{2}\right)\overset{N}{\underset{i=1}{\sum }}\overset{N}{\underset{j=1}{\sum }}G(y_{i}-y_{j},2\sigma^{2}I)$$

$${\text{V}}_{\text{BTW}}=\frac{1}{N^{3}}\overset{M}{\underset{c=1}{\sum }}J_{c}\overset{N}{\underset{i=1}{\sum }}\overset{J_{c}}{\underset{j=1}{\sum }}G(y_{i}-y_{cj},2\sigma^{2}I)$$ (8)

Mutual Information Bias. Bias can be estimated on the basis of an approximate formula given in Panzeri et al. [71] eq 4:

$$BIAS\left[MI\left(C,S\right)\right]\sim \left(1/N\right)\left\{\underset{s}{\sum }\left(\bar{R_{s}}-1\right)-\left(\bar{R}-1\right)\right\}$$ (9)

where $\overset{-}{R_{s}}$ denotes the number of relevant responses for the stimulus conditional response probability distribution *p*(*C*|*S*) and $\overset{-}{R}$ denotes the number of relevant responses for *p*(*C*), i.e. the number of different responses C with nonzero probability of being observed across all stimuli. *S* is one-dimensional stimulus projection (considered here for simplicity). Now $\overset{-}{R}$ does not depend on the frame size *r*, but $\overset{-}{R_{s}}$ does because the conditional probability distribution *p*(*C*|*S*) can change with *r*. To explain this let us use a very simple case of two input vectors, one that produces a spike (*C* = 1) and one that causes no spikes (*C* = 0). Let us assume that for a small *r* the projection values are *S*(spike) = 1 and *S*(nospike) = −1, and for large *r*: *S*(spike) = 10 and *S*(nospike) = −10. The conditional probability distributions (which have Gaussian shape and let assume σ = 2) will be *p*(*C* = 1|*S* = −10) = 0 and *p*(*C* = 0|*S* = −10) = 0, but will have non-negligible values for *p*(*C* = 1|*S* = −1) and *p*(*C* = 0|*S* = −1). Therefore $\overset{-}{R_{s}}$ will be bigger for smaller *r* and hence the bias, as per eq (9), if σ is constant.

Receptive Field Vectors and error estimation. A standard jackknife technique was used [77, 78]: We first split the dataset into *N*_jack_ datasets and use *N*_jack_-1 to calculate the RFVs, by omitting one dataset at a time. This resulted in *N*_jack_ estimates of the vectors and the final result was the average across these estimates. They were also used to find the standard error. In calculating the error we used the jackknife estimate of the standard error from ref.[78] eq (10.34). The estimation is due to the work of John Tuckey in the late 1950's and it has a factor (n-1)/n in the square root instead of the usual 1/[n(n-1)] for the standard deviation. More details about the implementation of the method can be found in [34].

#### Predictive power of the model

Using the RFVs it is possible to predict responses of RGCs to a variety of inputs using a common model such as the Linear-Nonlinear-Poisson cascade model [12, 16, 17]. Results for the predictive power of the reduced stimulus space and an analysis of the effects of nonlinearity [2, 12, 63, 79] that can be captured by this approach will be published soon.

#### Spot Stimulus–Analysis of Physiological Data

The Spot stimulus consisted of two types of stimulus protocols (presented at 3.75 μm/pixel via the projector):

1. Expanding Black Spot (BS): frames alternating between uniform gray and gray with a black spot of diameters 125, 250, 375, 500, 625 and 1250 μm. Every gray frame is presented for 3 seconds (the initial one for 2 seconds) and then a frame with a black spot is presented for 2 seconds, ref. [7] Fig 2.
2. Expanding White Spot (WS): exactly the same protocol as with the BS but with a white spot,

The light intensity contrast was K = 1.86 (black to gray) and 1.88 (gray to white), i.e. the ratio of intensities was approximately B:G:W = 1:2:4. The gray background was in photopic region.

- Firing Rate (*F*): peri-stimulus time histogram (time bin size 10ms) or Parzen window density estimation, we use Gaussian kernel of the width *σ* [76].
- Total number of spikes (*N*): the number of spikes during a 2 second cycle for the spot stimulation.
- Cell Response (*N*_*max*_): the total number of spikes in the cycle at which this is at maximum.
- Transient/Sustained firing measures: assuming an exponential decrease of the firing rate after the peak, the relative firing rate (*f* = *F*/*F*_*max*_) can be approximated with: *f* = *f*_*s*_ + (1 − *f*_*s*_)·*exp*(−*t*/*τ*), where τ is the time constant of the firing rate drop, and *f*_*s*_ is the “steady state” relative firing rate which corresponds to the sustained firing rate towards the end of the stimulus (2 seconds).
- Surround-Inhibition-Ratio: *SIR* = 1 − *N*(*D*_*max*_)/*N*_*max*_, where *N*(*D*_*max*_)/*N*_*max*_ is the ratio of the total number of spikes for the maximum spot diameter (*D*_*max*_ = 1250μm) and cell’s maximum response (*N*_*max*_).
- (Static) Spatial Contrast index: *SCI =* 1 − *N*_*max*_(*spatial contrast ON*)/*N*_*max*_(*spatial contrast OFF*), compares the cell’s spiking Response for the same temporal contrast but with and without spatial contras, e.g. response to BS-ON vs. WS-OFF (nominally the same temporal contrast, but the first creates the static spatial contrast, the second removes it).
- Latency time: the time interval between the onset of a stimulus and the peak of the firing rate. The firing rate distribution function was constructed using the Gaussian Parzen-windows, = 8ms.
- Receptive Field of optimal response for the Spot Stimulus: The positions of the maximum on the cubic spline interpolation diagram *N* vs *D* and *FR* vs *D* are taken as indicators for the size of the stimulus spot that has the strongest effect on the cell’s response.

## Supporting Information

S1 Fig Morphological quantification of PV RGCs into eight groups. (a) Maximum intensity confocal microscopic projections of representative neurobiotin-labeled PV cells PV0-PV7. Note electrical coupling of PV5 (5 ganglion cell somata) and PV7 (4 small somata within dendritic field) via diffusion of neurobiotin through gap junctions. Scale bars, 50 μm. (b) Vertical section of the retina immunoreacted for calretinin (magenta) and ChAT (cyan) revealing inner plexiform layer (IPL) strata. DAPI (gray) labels cell nuclei. ONL, outer nuclear layer; OPL, outer plexiform layer; INL, inner nuclear layer. (b) Percent dendritic depth of 182 PV cells (black bars; mean ± SEM) relative to the ChAT bands (gray boxes: -15 to 15% for ON ChAT band, 85 to 115% for OFF ChAT band; middle box is the middle calretinin band, 30 to 60%). Bistratified cells are shown on the right (two bars per cell). (c-top) Two-dimensional cluster of PV cells (n = 182) for k = 8 clusters. Each cluster corresponds to a different cell type (see Results): PV7 –dark blue, PV6 –yellow, PV5 –magenta, PV4 –cyan, PV3 –red, PV2 –green, PV1 –teal. Bistratified cells are black (PV0); each point is from a pair. Y-axis, the depth range is plotted between the mean GCL (-136%) and INL (202%) borders.; x-axis, dendritic field area. (c-bottom) Mean (black points) and standard deviation (dark gray boxes) of each cluster from (c-top), including both strata from bistratifed cells at 0% and 100% depth. Marker bands are light-gray. Modified from [7] with permission.
(PDF)

S2 Fig Visual stimuli. (a) Natural scene, frames 320x240 pixels usually displayed for 40ms (25 fps). For details of light stimulation parameters and contrast see ref. [8]. (b) Average spatial correlation within frames, (c) Average temporal correlation from frame to frame (502 frames in total).
(PDF)

S3 Fig Visual response for PV1 cells to the natural stimulus sequence. The movies are labelled catMov1, catMov2 and cat Mov3 –described above in S2 Fig. The onset of movies is at 0, and the movies last for 142 (catMov1), 189 (catMov2) and 174 (catMov1) frames. Before and after the movies the retina is exposed to the uniform gray light. Different cells are shown in alternating red and blue colours. Within each colour group each row is an individual recording. Recordings for 11 cells, for each cell trials repeated 4–18 times.
(PDF)

S4 Fig Raster plots for PV5 cells response to natural scene movies. Recordings for 7 cells are shown, for each cell trials are repeated 4–10 times.
(PDF)

S5 Fig (a) A single RFV for a PV0 cell, but with the response (weights) taken to be proportional to the product of the number of spikes in two successive bins instead of just the number of spikes. In this way bursts of spikes are better represented. Although this approach has some similarities to the method which identifies the relevant variables as quadratic forms (“stimulus energies”) as in [79], it is more related to event spike triggered analysis described by de Ruyter van Steveninck and Bialek [21] and analysis about the information carried by compound events in spike trains (such as spike bursts) by Brenner *et al* [23]. (b) The two vectors for a PV5 cell when the outputs were separated into three classes. The classes are, C0: no spikes (nS = 0, blue), C1: average number of spikes between 0 and 1 (0<nS<1, green), and C2: more than 2 spikes (nS>2, red). (c) One-dimensional and (d) two-dimensional plots of the separation of the input stimuli on the basis of their projections onto ***w***_1_ (Projection 1) and ***w***_2_ (Projection 2) and the number of spikes in the projection. With reference to ***w***_1_: Class C0 is negatively correlated, C1 is positively correlated and C2 is not dependent (projection values are near zero). With reference to ***w***_2_: C2 is positively correlated, so will occur when RFV2 is seen by the cell, C2 is negatively correlated, so they occur when the inverse of RFV1 is seen, and C1 moderately negatively correlated.
(PDF)

S6 Fig RFVs for multiple cells of the same class. (top) Table shows a summary of the recordings for seven different cells from the PV5 class. Columns are: cell number, cell name, number of frames per second for the natural scene movies, time period for each frame, number of recordings for each movie (catMov1 has 141 frames, catMov2 has 188 frames and catMov3 has 173 frames), number of frame periods with zero spikes, number of frame periods with spikes–the mean number of spikes per period is: less than 0.5, between 0.5 and 2, and more than 2, and total, and finaly the total number of spikes for the complete natural scene stimulus consisting of 502 frames. (bottom) RFVs for each cell. Note that the cell 1 has slower changing stimulus (11 frames per second) and correspondingly the RFV is only 4 frames long (corresponds to 4x92ms ≈ 370ms).
(PDF)

S1 Table Physiological Properties of the PV RGCs. BS–Black Spot, WS–White Spot, NatS–Natural Stimulus, Nc–number of cells (3–9 recordings per cell per stimulus).
(PDF)

## References

[1] Wassle H. Parallel processing in the mammalian retina. Nat Rev Neurosci. 2004;5(10):747–57. 15378035 10.1038/nrn1497

[2] Gollisch T, Meister M. Rapid Neural Coding in the Retina with Relative Spike Latencies. Science. 2008;319(5866):1108–11. 10.1126/science.1149639 18292344

[3] Masland RH. The fundamental plan of the retina. Nat Neurosci. 2001;4(9):877–86. 11528418 10.1038/nn0901-877

[4] Roska B, Molnar A, Werblin FS. Parallel processing in retinal ganglion cells: how integration of space-time patterns of excitation and inhibition form the spiking output. J Neurophysiology. 2006;95(6):3810–22. 16510780 10.1152/jn.00113.2006

[5] Roska B, Werblin F. Vertical interactions across ten parallel, stacked representations in the mammalian retina. Nature. 2001;410(6828):583–7. 11279496 10.1038/35069068

[6] Azeredo da Silveira R, Roska B. Cell Types, Circuits, Computation. Current Opinion in Neurobiology. 2011;21(5):664–71. 10.1016/j.conb.2011.05.007 21641794

[7] Farrow K, Teixeira M, Szikra T, Viney Tim J, Balint K, Yonehara K, et al Ambient Illumination Toggles a Neuronal Circuit Switch in the Retina and Visual Perception at Cone Threshold. Neuron. 2013;78(2):325–38. 10.1016/j.neuron.2013.02.014 23541902

[8] Muench T, da Silveira R, Siegert S, Viney T, Awatramani G, Roska B. Approach Sensitivity in the Retina Processed by a Multifunctional Neural Circuit. Nature Neuroscience. 2009;10:1308–16. 10.1038/nn.2389 19734895

[9] Betsch BY, Einhauser W, Kording KP, Konig P. The world from a cat's perspective—statistics of natural videos. Biol Cybern. 2004;90(1):41–50. 14762723 10.1007/s00422-003-0434-6

[10] Kayser C, Salazar RF, Konig P. Responses to natural scenes in cat V1. J Neurophysiol. 2003;90(3):1910–20. 12750423 10.1152/jn.00195.2003

[11] Smyth D, Willmore B, Baker GE, Thompson ID, Tolhurst DJ. The Receptive-Field Organization of Simple Cells in Primary Visual Cortex of Ferrets under Natural Scene Stimulation. J Neuroscience. 2003;23(11):4746–59. 12805314 10.1523/JNEUROSCI.23-11-04746.2003 PMC6740783

[12] Chichilnisky EJ. A simple white noise analysis of neuronal light responses. Network: Computation in Neural Systems. 2001;12(2):199–213. 11405422

[13] Steveninck RDRV, Bialek W. Reliability and Statistical Efficiency of a Blowfly Movement-Sensitive Neuron. Philosophical Transactions of the Royal Society of London Series B: Biological Sciences. 1995;348(1325):321–40.

[14] Dayan P, Abbott LF. Theoretical neuroscience: Computational and mathematical modeling of neural systems. Cambridge: MIT Press; 2001.

[15] Schwartz O, Pillow JW, Rust NC, Simoncelli EP. Spike-triggered neural characterization. Journal of Vision. 2006;6(4). 10.1167/6.4.13 16889482

[16] Paninski L. Convergence properties of three spike-triggered analysis techniques. Network (Bristol, England). 2003;14(3):437–64. 12938766

[17] Pillow JW, Simoncelli EP. Dimensionality reduction in neural models: An information-theoretic generalization of spike-triggered average and covariance analysis. Journal of Vision. 2006;6(4). 10.1167/6.4.9 16889478

[18] Bomash I, Roudi Y, Nirenberg S. A Virtual Retina for Studying Population Coding. PLoS ONE. 2013;8(1):e53363 10.1371/journal.pone.0053363 23341940 PMC3544815

[19] Nirenberg S, Pandarinath C. Retinal prosthetic strategy with the capacity to restore normal vision. Proceedings of the National Academy of Sciences. 2012;109(37):15012–7. 10.1073/pnas.1207035109 PMC3443127 22891310

[20] Paninski L, Pillow J, Lewi J. Statistical models for neural encoding, decoding, and optimal stimulus design In: Paul Cisek TD, John FK, editors. Progress in Brain Research. Volume 165: Elsevier; 2007 p. 493–507. 10.1016/S0079-6123(06)65031-0 17925266

[21] De Ruyter Van Steveninck R, Bialek W. Real-time Performance of a Movement-Sensitive Neuron in the Blowfly Visual System: Coding and Information Transfer in Short Spike Sequences. Proceedings of the Royal Society of London—Biological Sciences. 1988;234(1277):379–414.

[22] Dimitrov A, Lazar A, Victor J. Information theory in neuroscience. J Comput Neurosci. 2011;30(1):1–5. 10.1007/s10827-011-0314-3 21279429 PMC3736735

[23] Brenner N, Strong SP, Koberle R, Bialek W, Steveninck RRdRv. Synergy in a Neural Code. Neural Computation. 2000;12(7):1531–52. 10935917 10.1162/089976600300015259

[24] Wu MCK, David SV, Gallant JL. Complete Functional Characterization of Sensory Neurons by System Identification. Annual Review of Neuroscience. 2006;29(1):477–505. 10.1146/annurev.neuro.29.051605.113024 16776594

[25] Sharpee T, Rust NC, Bialek W. Analyzing neural responses to natural signals: maximally informative dimensions. Neural Comput. 2004;16(2):223–50. 15006095 10.1162/089976604322742010

[26] Fitzgerald JD, Rowekamp RJ, Sincich LC, Sharpee TO. Second Order Dimensionality Reduction Using Minimum and Maximum Mutual Information Models. PLoS Comput Biol 2011;7(10):e1002249 10.1371/journal.pcbi.1002249 22046122 PMC3203063

[27] Torkkola K. Feature extraction by non parametric mutual information maximization. J Mach Learn Res. 2003;3:1415–38.

[28] Xu D, Erdogmuns D. Renyi’s Entropy, Divergence and Their Nonparametric Estimators In: Principe JC, editor. Information Theoretic Learning: Springer New York; 2010 p. 47–102.

[29] MacKay D. Information Theory, Inference, and Learning Algorithms. Cambridge University Press; 2003 640 p.

[30] Renyi A, editor On Measures of Entropy and Information Proceedings of the Fourth Berkeley Symposium on Mathematical Statistics and Probability, Volume 1: Contributions to the Theory of Statistics; 1961; Berkeley, Calif: University of California Press.

[31] Kapur JN. Measures of Information and Their Applications: Wiley, New Delhi, India; 1994.

[32] Principe JC, Xu D, Fisher JW III. Information Theoretic Learning, in Unsupervised Adaptive Filtering. In: Haykin S, editor. New York: Wiley; 2000 p. 265–319.

[33] Kouh M, Sharpee TO. Estimating linear–nonlinear models using Rényi divergences. Network: Computation in Neural Systems. 2009;20(2):49–68. 10.1080/09548980902950891 PMC2782376 19568981

[34] Rowekamp RJ, Sharpee TO. Analyzing multicomponent receptive fields from neural responses to natural stimuli. Network: Computation in Neural Systems. 2011;22(1–4):45–73. 10.3109/0954898X.2011.566303 PMC3251001 21780916

[35] Jeon CJ, Strettoi E, Masland RH. The major cell populations of the mouse retina. J Neurosci. 1998;18(21):8936–46. 9786999 10.1523/JNEUROSCI.18-21-08936.1998 PMC6793518

[36] MacNeil MA, Masland RH. Extreme Diversity among Amacrine Cells: Implications for Function. Neuron. 1998;20(5):971–82. 9620701 10.1016/s0896-6273(00)80478-x

[37] Victor JD. Analyzing receptive fields, classification images and functional images: challenges with opportunities for synergy. Nat Neurosci. 2005;8(12):1651–6. 16306893 10.1038/nn1607 PMC1622929

[38] Kim IJ, Zhang Y, Yamagata M, Meister M, Sanes JR. Molecular identification of a retinal cell type that responds to upward motion. Nature. 2008;452(7186):478–82. Epub 2008/03/28. 10.1038/nature06739 18368118

[39] Werblin FS, Dowling JE. Organization of the retina of the mudpuppy, Necturus maculosus. II. Intracellular recording. Journal of neurophysiology. 1969;32(3):339–55. 4306897 10.1152/jn.1969.32.3.339

[40] Yonehara K, Farrow K, Ghanem A, Hillier D, Balint K, Teixeira M, et al The First Stage of Cardinal Direction Selectivity Is Localized to the Dendrites of Retinal Ganglion Cells. Neuron. 2013;79(6):1078–85. 10.1016/j.neuron.2013.08.005 23973208

[41] Barlow HB, Hill RM, Levick WR. Retinal ganglion cells responding selectively to direction and speed of image motion in the rabbit. Journal of Physiology. 1964;174(3):377–407. 10.1113/jphysiol.1964.sp007463 PMC1368915 14220259

[42] Rockhill RL, Daly FJ, MacNeil MA, Brown SP, Masland RH. The Diversity of Ganglion Cells in a Mammalian Retina. The Journal of Neuroscience. 2002;22(9):3831–43. 11978858 10.1523/JNEUROSCI.22-09-03831.2002 PMC6758366

[43] Elstrott J, Feller MB. Vision and the establishment of direction-selectivity: a tale of two circuits. Current Opinion in Neurobiology. 2009;19(3):293–7. 10.1016/j.conb.2009.03.004 19386483 PMC2805110

[44] Chan Y-C, Chiao C-C. The distribution of the preferred directions of the ON–OFF direction selective ganglion cells in the rabbit retina requires refinement after eye opening. Physiological Reports. 2013;1(2):e00013 10.1002/phy2.13 24303104 PMC3831909

[45] Wassle H, Boycott BB. Functional architecture of the mammalian retina. Physiological Reviews. 1991;71(2):447–80. 2006220 10.1152/physrev.1991.71.2.447

[46] Sun W, Li N, He S. Large-scale morphological survey of mouse retinal ganglion cells. J. Comparative Neurology. 2002;451(2):115–26. 10.1002/cne.10323 12209831

[47] Pang J-J, Gao F, Wu SM. Light-Evoked Excitatory and Inhibitory Synaptic Inputs to ON and OFF α Ganglion Cells in the Mouse Retina. J Neuroscience. 2003;23(14):6063–73. 12853425 10.1523/JNEUROSCI.23-14-06063.2003 PMC6740343

[48] Remtulla S, Hallett PE. A schematic eye for the mouse, and comparisons with the rat. Vision Res. 1985;25(1):21–31. 3984214 10.1016/0042-6989(85)90076-8

[49] Famiglietti EV. Synaptic organization of complex ganglion cells in rabbit retina: Type and arrangement of inputs to directionally selective and local-edge-detector cells. The Journal of Comparative Neurology. 2005;484(4):357–91. 15770656 10.1002/cne.20433

[50] Zhang Y, Kim I-J, Sanes JR, Meister M. The most numerous ganglion cell type of the mouse retina is a selective feature detector. Proceedings of the National Academy of Sciences. 2012. 10.1073/pnas.1211547109 PMC3437843 22891316

[51] Roska B, Werblin F. Rapid global shifts in natural scenes block spiking in specific ganglion cell types. Nat Neurosci. 2003;6(6):600–8. 12740583 10.1038/nn1061

[52] Trong PK, Rieke F. Origin of correlated activity between parasol retinal ganglion cells. Nat Neurosci. 2008;11(11):1343–51. 10.1038/nn.2199 18820692 PMC2575139

[53] Schwartz G, Rieke F. Nonlinear spatial encoding by retinal ganglion cells: when 1 + 1 ≠ 2. J General Physiology. 2011;138(3):283–90. 10.1085/jgp.201110629 PMC3171084 21875977

[54] Kong J-H, Fish DR, Rockhill RL, Masland RH. Diversity of ganglion cells in the mouse retina: Unsupervised morphological classification and its limits. J Comparative Neurology. 2005;489(3):293–310. 10.1002/cne.20631 16025455

[55] Coombs J, van der List D, Wang GY, Chalupa LM. Morphological properties of mouse retinal ganglion cells. Neuroscience. 2006;140(1):123–36. 16626866 10.1016/j.neuroscience.2006.02.079

[56] Zeck GM, Masland RH. Spike train signatures of retinal ganglion cell types. European Journal of Neuroscience. 2007;26(2):367–80. 17650112 10.1111/j.1460-9568.2007.05670.x

[57] Farrow K, Masland RH. Physiological clustering of visual channels in the mouse retina. Journal of Neurophysiology. 2011;105(4):1516–30. 10.1152/jn.00331.2010 21273316 PMC3075295

[58] Kim T-J, Jeon C-J. Morphological Classification of Parvalbumin-Containing Retinal Ganglion Cells in Mouse: Single-Cell Injection after Immunocytochemistry. Investigative Ophthalmology & Visual Science. 2006;47(7):2757–64. 16799011 10.1167/iovs.05-1442

[59] Lee E-S, Lee J-Y, Jeon C-J. Types and density of calretinin-containing retinal ganglion cells in mouse. Neuroscience Research. 2010;66(2):141–50. 10.1016/j.neures.2009.10.008 19895859

[60] Huberman AD, Wei W, Elstrott J, Stafford BK, Feller MB, Barres BA. Genetic Identification of an On-Off Direction- Selective Retinal Ganglion Cell Subtype Reveals a Layer-Specific Subcortical Map of Posterior Motion. Neuron. 2009;62(3):327–34. 10.1016/j.neuron.2009.04.014 19447089 PMC3140054

[61] Zhu Y, Xu J, Hauswirth WW, DeVries SH. Genetically Targeted Binary Labeling of Retinal Neurons. J Neuroscience. 2014;34(23):7845–61. 10.1523/JNEUROSCI.2960-13.2014 24899708 PMC4044247

[62] Greschner M, Shlens J, Bakolitsa C, Field GD, Gauthier JL, Jepson LH, et al Correlated firing among major ganglion cell types in primate retina. The Journal of Physiology. 2011;589(1):75–86. 20921200 10.1113/jphysiol.2010.193888 PMC3039261

[63] Schwartz GW, Okawa H, Dunn FA, Morgan JL, Kerschensteiner D, Wong RO, et al The spatial structure of a nonlinear receptive field. Nat Neurosci. 2012;15(11):1572–80. 10.1038/nn.3225 23001060 PMC3517818

[64] Grimes William N, Schwartz Gregory W, Rieke F. The Synaptic and Circuit Mechanisms Underlying a Change in Spatial Encoding in the Retina. Neuron. 2014;82(2):460–73. 10.1016/j.neuron.2014.02.037 24742466 PMC4038266

[65] Chen SK, Badea TC, Hattar S. Photoentrainment and pupillary light reflex are mediated by distinct populations of ipRGCs. Nature. 2011;476(7358):92–5. 10.1038/nature10206 21765429 PMC3150726

[66] Schmidt Tiffany M, Alam Nazia M, Chen S, Kofuji P, Li W, Prusky Glen T, et al A Role for Melanopsin in Alpha Retinal Ganglion Cells and Contrast Detection. Neuron. 2014;82(4):781–8. 10.1016/j.neuron.2014.03.022 24853938 PMC4083763

[67] Sekaran S, Lupi D, Jones SL, Sheely CJ, Hattar S, Yau KW, et al Melanopsin-Dependent Photoreception Provides Earliest Light Detection in the Mammalian Retina. Current Biology. 2005;15(12):1099–107. 15964274 10.1016/j.cub.2005.05.053 PMC4316668

[68] Melyan Z, Tarttelin EE, Bellingham J, Lucas RJ, Hankins MW. Addition of human melanopsin renders mammalian cells photoresponsive. Nature. 2005;433(7027):741–5. 15674244 10.1038/nature03344

[69] Soo FS, Schwartz GW, Sadeghi K, Berry MJ. Fine Spatial Information Represented in a Population of Retinal Ganglion Cells. J Neuroscience. 2011;31(6):2145–55. 10.1523/JNEUROSCI.5129-10.2011 21307251 PMC3526660

[70] Nirenberg S, Carcieri SM, Jacobs AL, Latham PE. Retinal ganglion cells act largely as independent encoders. Nature. 2001;411(6838):698–701. 11395773 10.1038/35079612

[71] Panzeri S, Senatore R, Montemurro MA, Petersen RS. Correcting for the Sampling Bias Problem in Spike Train Information Measures. J Neurophysiology. 2007;98(3):1064–72. 17615128 10.1152/jn.00559.2007

[72] Simoncelli EP, Olshausen BA. Natural Image Statistics and Neural Representation. Annual Review of Neuroscience. 2001;24(1):1193–216. 10.1146/annurev.neuro.24.1.1193 11520932

[73] Quiroga RQ, Nadasdy Z, Ben-Shaul Y. Unsupervised spike detection and sorting with wavelets and superparamagnetic clustering. Neural Comput. 2004;16(8):1661–87. 15228749 10.1162/089976604774201631

[74] Nishimori Y, Akaho S. Learning algorithms utilizing quasi-geodesic flows on the Stiefel manifold. Neurocomput. 2005;67:106–35.

[75] Wen Z, Yin W. A Feasible method for Optimization with Orthogonality Constraints. Rice University 2010:1–34.

[76] Parzen E. On the estimation of probability density function and the mode. Annals of Mathematical Statistics. 1962;33(3):1065–76.

[77] Efron B, Tibshirani R. Bootstrap Methods for Standard Errors, Confidence Intervals, and Other Measures of Statistical Accuracy. Statist Sci. 1986;1(1):54–75.

[78] Efron B, Tibshirani R. An Introduction to the Bootstrap: Chapman & Hall/CRC; 1993.

[79] Rajan K, Bialek W. Maximally Informative "Stimulus Energies" in the Analysis of Neural Responses to Natural Signals. PLoS ONE. 2013;8(11):e71959 10.1371/journal.pone.0071959 24250780 PMC3826732

