# Supplementary figures and images for: Receptive Field Vectors of Genetically-Identified Retinal Ganglion Cells Reveal Cell-Type-Dependent Visual Functions
Source: PLoS One. 2016 Feb 4;11(2):e0147738. doi: 10.1371/journal.pone.0147738 (PMC4742227; doi:10.1371/journal.pone.0147738)

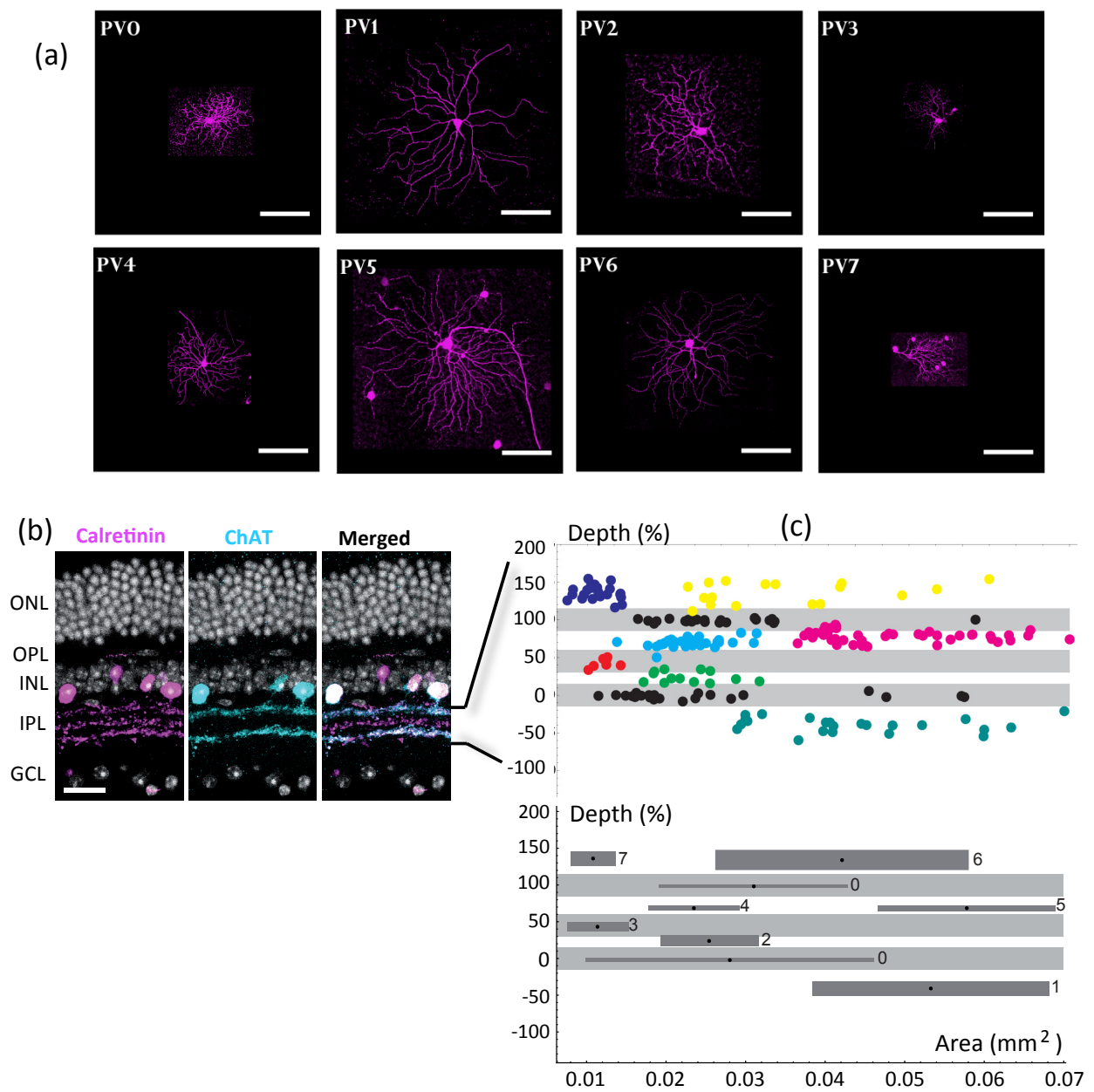

S1 Fig

Supplement: S1 Fig — (a) Maximum intensity confocal microscopic projections of representative neurobiotin-labeled PV cells PV0-PV7. Note electrical coupling of PV5 (5 ganglion cell somata) and PV7 (4 small somata within dendritic field) via diffusion of neurobiotin through gap junctions. Scale bars, 50 μm. (b) Vertical section of the retina immunoreacted for calretinin (magenta) and ChAT (cyan) revealing inner plexiform layer (IPL) strata. DAPI (gray) labels cell nuclei. ONL, outer nuclear layer; OPL, outer plexiform layer; INL, inner nuclear layer. (b) Percent dendritic depth of 182 PV cells (black bars; mean ± SEM) relative to the ChAT bands (gray boxes: -15 to 15% for ON ChAT band, 85 to 115% for OFF ChAT band; middle box is the middle calretinin band, 30 to 60%). Bistratified cells are shown on the right (two bars per cell). (c-top) Two-dimensional cluster of PV cells (n = 182) for k = 8 clusters. Each cluster corresponds to a different cell type (see Results): PV7 –dark blue, PV6 –yellow, PV5 –magenta, PV4 –cyan, PV3 –red, PV2 –green, PV1 –teal. Bistratified cells are black (PV0); each point is from a pair. Y-axis, the depth range is plotted between the mean GCL (-136%) and INL (202%) borders.; x-axis, dendritic field area. (c-bottom) Mean (black points) and standard deviation (dark gray boxes) of each cluster from (c-top), including both strata from bistratifed cells at 0% and 100% depth. Marker bands are light-gray. Modified from [7] with permission. (PDF) [file pone.0147738.s001.pdf]

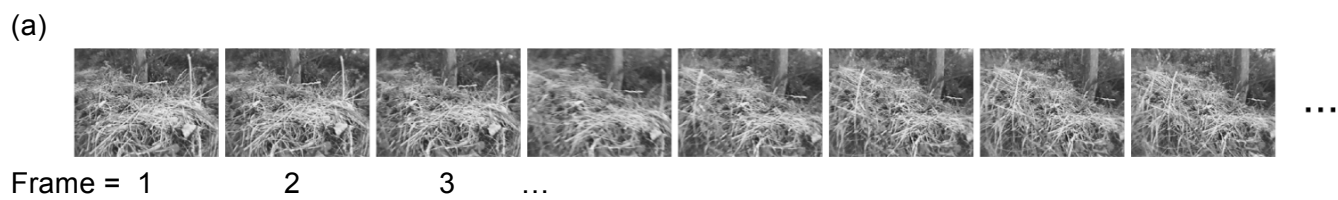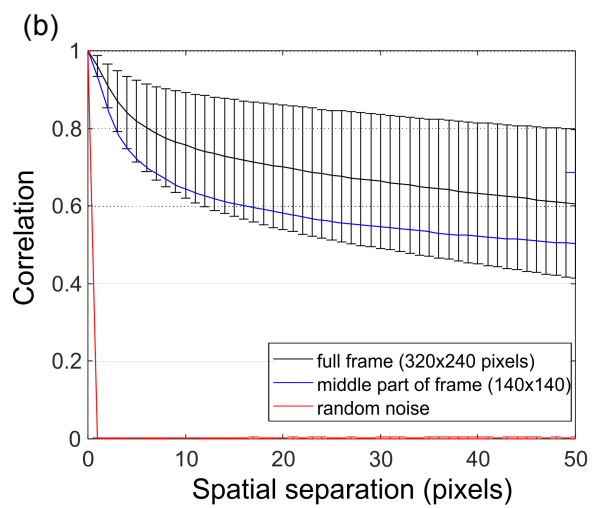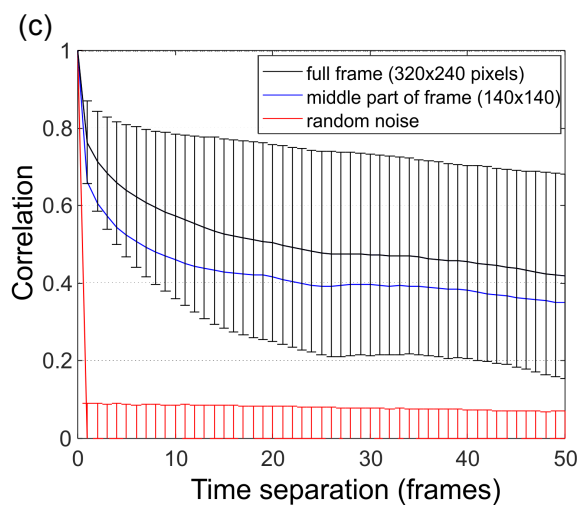

S2 Fig.

Supplement: S2 Fig — (a) Natural scene, frames 320x240 pixels usually displayed for 40ms (25 fps). For details of light stimulation parameters and contrast see ref. [8]. (b) Average spatial correlation within frames, (c) Average temporal correlation from frame to frame (502 frames in total). (PDF) [file pone.0147738.s002.pdf]

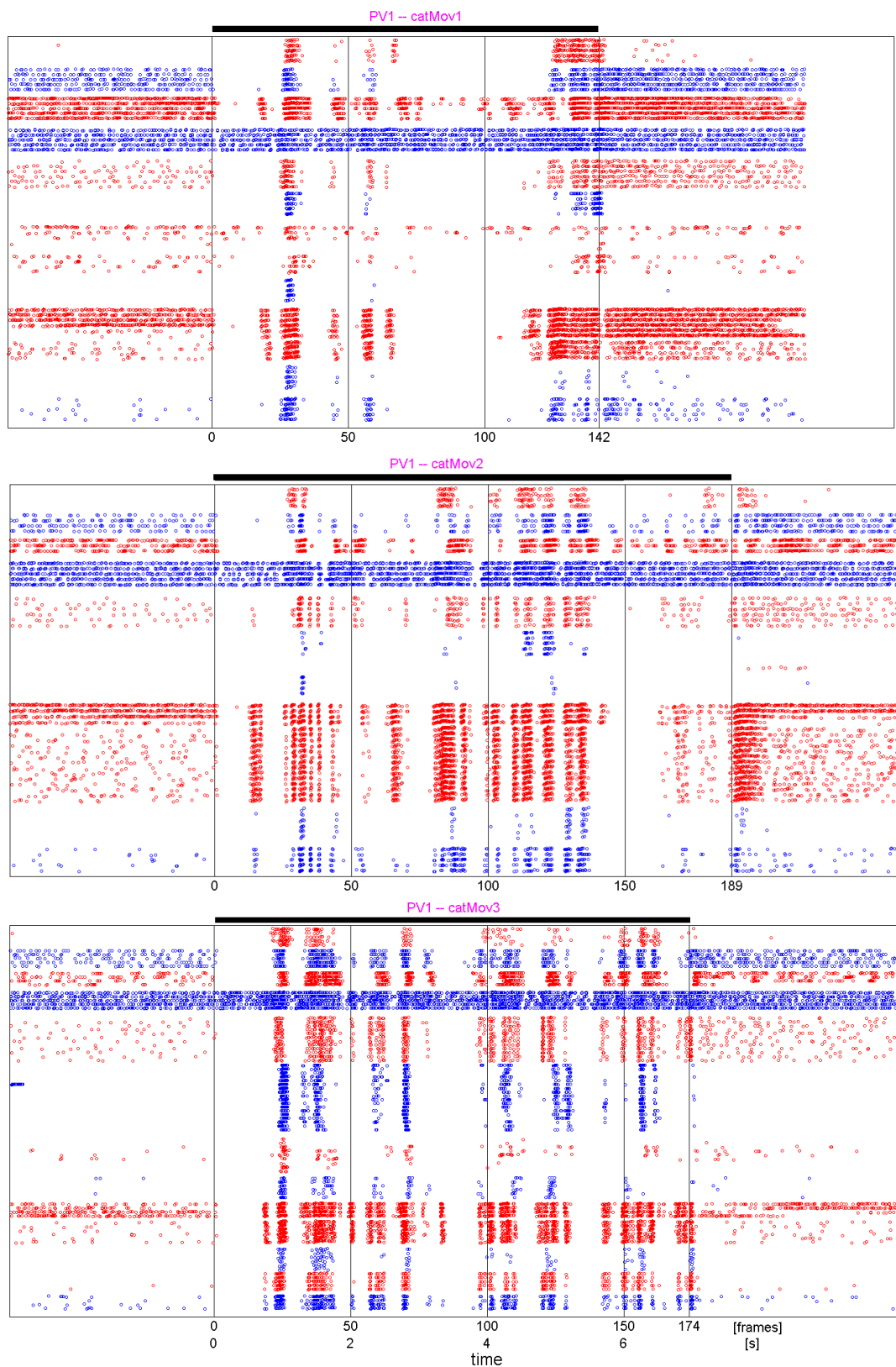

S3 Fig.

Supplement: S3 Fig — The movies are labelled catMov1, catMov2 and cat Mov3 –described above in S2 Fig. The onset of movies is at 0, and the movies last for 142 (catMov1), 189 (catMov2) and 174 (catMov1) frames. Before and after the movies the retina is exposed to the uniform gray light. Different cells are shown in alternating red and blue colours. Within each colour group each row is an individual recording. Recordings for 11 cells, for each cell trials repeated 4–18 times. (PDF) [file pone.0147738.s003.pdf]

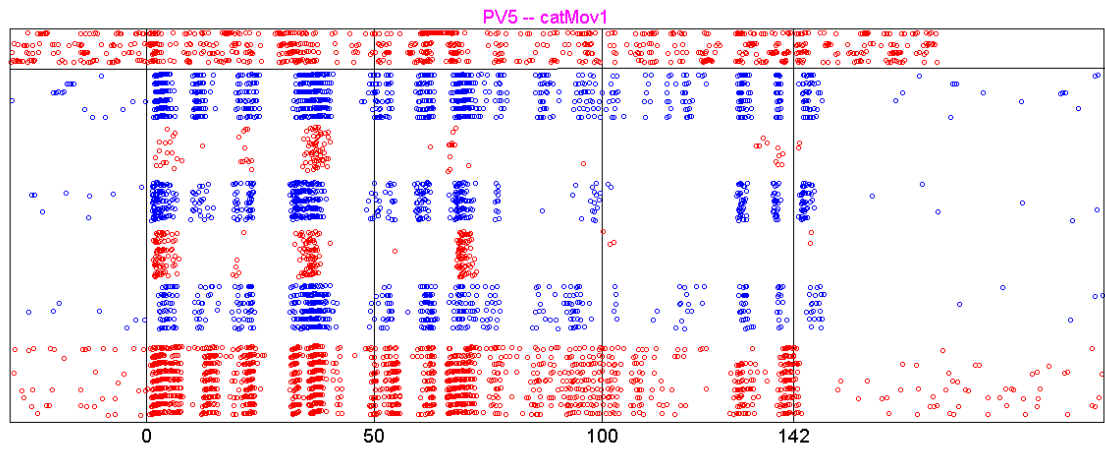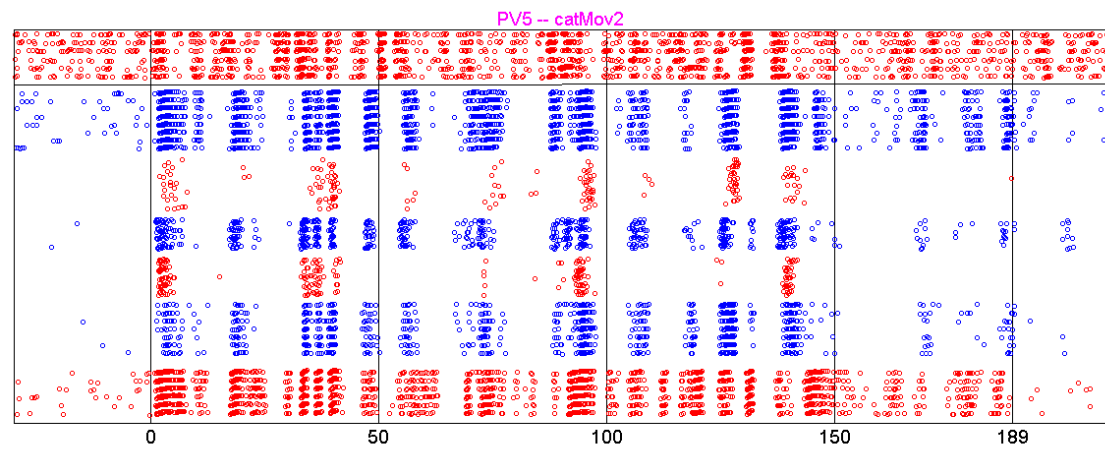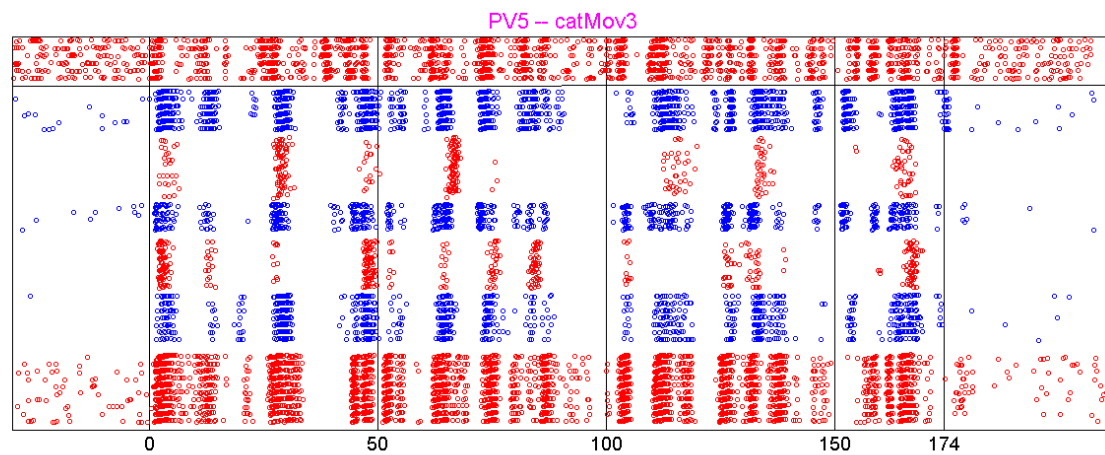

time [frames]: 100 frames = 4 sec, except first cell where 100 frames = 9.2 sec

S4 Fig.

Supplement: S4 Fig — Recordings for 7 cells are shown, for each cell trials are repeated 4–10 times. (PDF) [file pone.0147738.s004.pdf]

(a) PV0 cell, frame rate = 40ms, outer D=440 $\mu$ m.

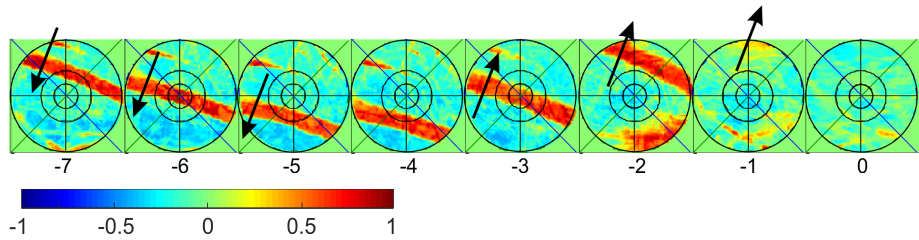

(b)

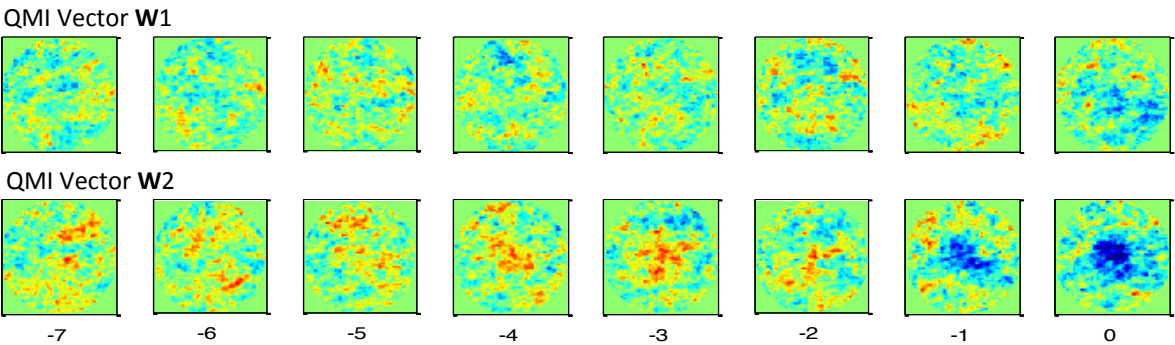

(c)

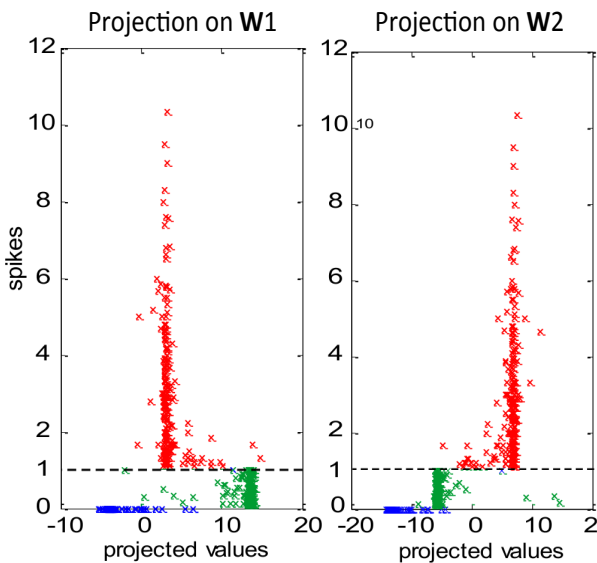

(d)

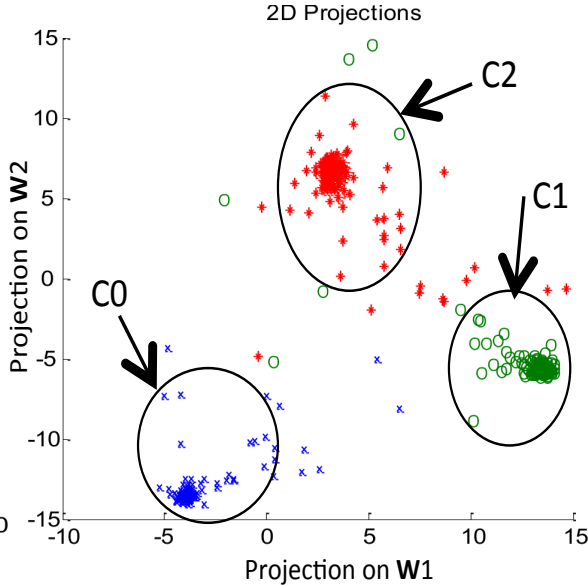

S5 Fig.

Supplement: S5 Fig — (a) A single RFV for a PV0 cell, but with the response (weights) taken to be proportional to the product of the number of spikes in two successive bins instead of just the number of spikes. In this way bursts of spikes are better represented. Although this approach has some similarities to the method which identifies the relevant variables as quadratic forms (“stimulus energies”) as in [79], it is more related to event spike triggered analysis described by de Ruyter van Steveninck and Bialek [21] and analysis about the information carried by compound events in spike trains (such as spike bursts) by Brenner et al [23]. (b) The two vectors for a PV5 cell when the outputs were separated into three classes. The classes are, C0: no spikes (nS = 0, blue), C1: average number of spikes between 0 and 1 (0<nS<1, green), and C2: more than 2 spikes (nS>2, red). (c) One-dimensional and (d) two-dimensional plots of the separation of the input stimuli on the basis of their projections onto w1 (Projection 1) and w2 (Projection 2) and the number of spikes in the projection. With reference to w1: Class C0 is negatively correlated, C1 is positively correlated and C2 is not dependent (projection values are near zero). With reference to w2: C2 is positively correlated, so will occur when RFV2 is seen by the cell, C2 is negatively correlated, so they occur when the inverse of RFV1 is seen, and C1 moderately negatively correlated. (PDF) [file pone.0147738.s005.pdf]
