# Supplementary material for: Receptive Field Vectors of Genetically-Identified Retinal Ganglion Cells Reveal Cell-Type-Dependent Visual Functions
Source: PLoS One. 2016 Feb 4;11(2):e0147738. doi: 10.1371/journal.pone.0147738 (PMC4742227; doi:10.1371/journal.pone.0147738)
Supplement: S6 Fig — (top) Table shows a summary of the recordings for seven different cells from the PV5 class. Columns are: cell number, cell name, number of frames per second for the natural scene movies, time period for each frame, number of recordings for each movie (catMov1 has 141 frames, catMov2 has 188 frames and catMov3 has 173 frames), number of frame periods with zero spikes, number of frame periods with spikes–the mean number of spikes per period is: less than 0.5, between 0.5 and 2, and more than 2, and total, and finaly the total number of spikes for the complete natural scene stimulus consisting of 502 frames. (bottom) RFVs for each cell. Note that the cell 1 has slower changing stimulus (11 frames per second) and correspondingly the RFV is only 4 frames long (corresponds to 4x92ms ≈ 370ms). (PDF) [file pone.0147738.s006.pdf]

	cell	frame rate [Hz]	t [ms]	Number of recordings Mov1	Mov2	Mov3	no-spikes frames	frames with spikes <0.5	0.5-2	>2sp	total	spikes total
1.	PV5_20091104_C4	11	92	4	6	6	54	133	214	101	448	649
2.	PV5_20091127_C2	25	40	6	8	6	187	95	120	100	315	563
3.	PV5_20100205_C5	25	40	5	6	8	385	62	45	10	117	91
4.	PV5_20100228_C3	25	40	5	4	4	268	56	93	85	234	488
5.	PV5_20100308_C5	25	40	6	5	7	397	43	38	24	105	144
6.	PV5_20100514_C4	25	40	6	7	7	233	95	121	53	269	318
7.	PV5_20100601_C1	25	40	9	6	10	114	113	150	125	388	706

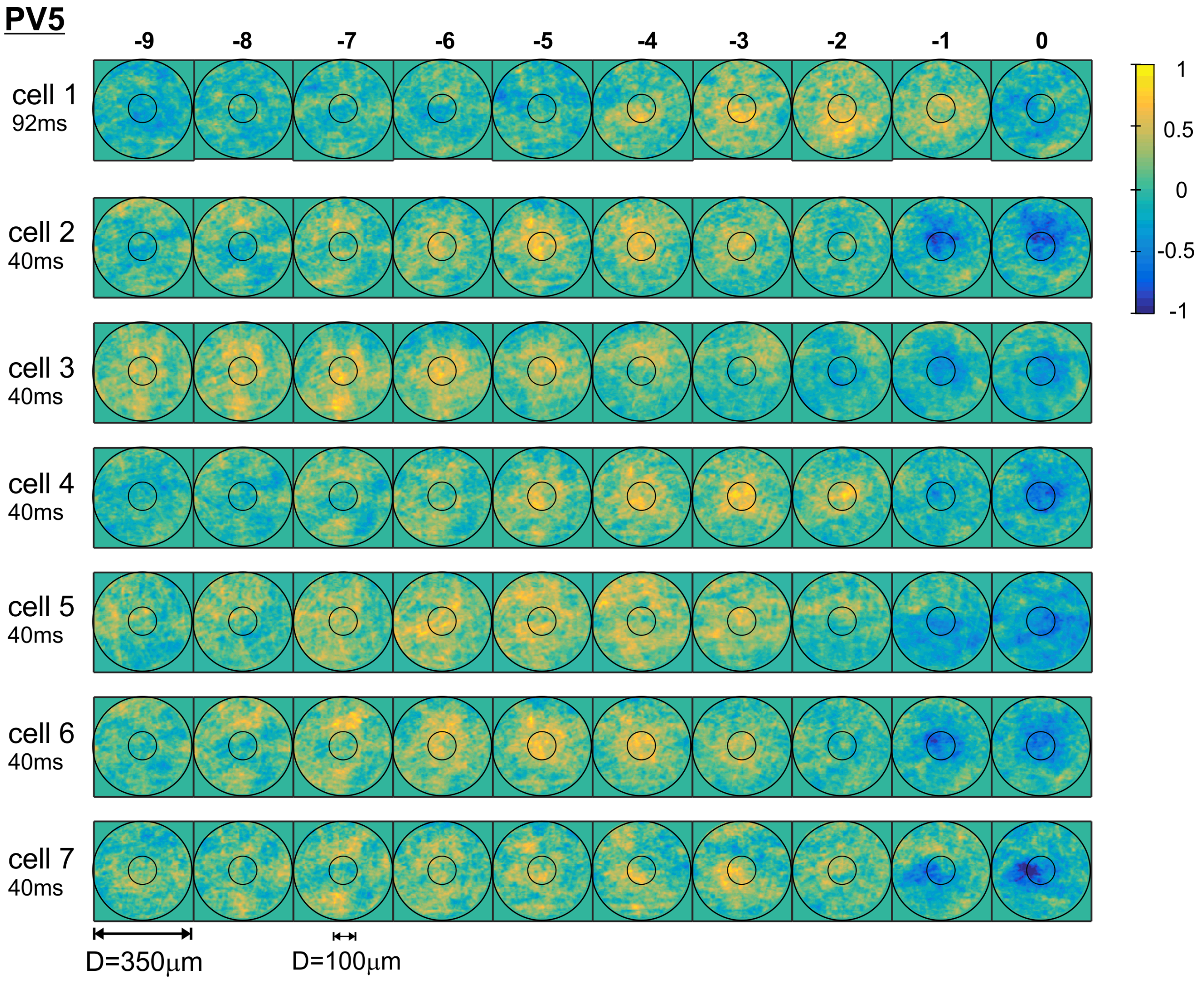

S6 Fig.
