# Supplementary material for: Receptive Field Vectors of Genetically-Identified Retinal Ganglion Cells Reveal Cell-Type-Dependent Visual Functions
Source: PLoS One. 2016 Feb 4;11(2):e0147738. doi: 10.1371/journal.pone.0147738 (PMC4742227; doi:10.1371/journal.pone.0147738)
Supplement: S1 Table — BS–Black Spot, WS–White Spot, NatS–Natural Stimulus, Nc–number of cells (3–9 recordings per cell per stimulus). (PDF) [file pone.0147738.s007.pdf]

			Total spikes in the max cycle		Firing Rate max cycle [Hz]		Transient or sustained		Surround Inhibition		Latency [ms]	Diameter [μm]		
												Dend Tree Field	Receptive Field estim	
			mean	median	mean	median	sustain	τ [ms]	peak	total	median		Spot	NatS
**PV7**	**OFF**	BS-On	5.4	4.7	141	133	0	44	100%	100%	83	118	<125	76
		WS-Off	2.5	1.5	102	100	0	10	100%	100%	95			
	**ON**	BS-Off	0.8	0.25	-	-	0	-	100%	100%	130		<125	
		WS-On	2.1	1	70	50	0	-	100%	100%	118			
		Nc=10	*low*		*low*		*sharp transient*		*complete*		*medium*	*small size*		
**PV6**	**OFF**	BS-On	90	71	244	167	20%	80	31%	30%	78	232	291	200
		WS-Off	44	42	144	160	7%	112	37%	23%	104		324	
	**ON**	BS-Off	10	4.3	-	-	-	-	-	-	-		-	
		WS-On	10	8.7	-	-	-	-	-	-	-		-	
		Nc=11	*low*		*low*		*sustained*		*weak*		*medium*	*large size*		
**PV5**	**OFF**	BS-On	37	33.1	472	480	<2%	45	44%	13%	70	271	389	220
		WS-Off	34	35.7	492	500	<1%	40	43%	31%	65		325	
	**ON**	BS-Off	*No response, only sometimes inhibition rebound after 500ms*											
		WS-On												
		Nc=12	*low*		*low*		*sharp transient*		*medium*		*fastest*	*large size*		
**PV4**	**OFF**	BS-On	27	27	265	250	~0%	65	81%	89%	74	173	178	96
		WS-Off	25	26.7	272	283	~0%	53	83%	94%	125		163	
	**ON**	BS-Off	*No response, only sometimes inhibition rebound*											
		WS-On												
		Nc=12/18	*low/med*		*medium*		*transient*		*strong*		*medslow*	*medium size*		
**PV3**	**OFF**	BS-On	56	37.5	121	113	~6%	224	100%	100%	250	121	<125	-
		WS-Off	38.5	35.8	122	125	~2%	200	100%	100%	332		<125	
	**ON**	BS-Off	10	9	76	71	-	-	100%	100%	300		<125	
		WS-On	20.3	14.9	133	150	-	-	100%	100%	312		<125	
		Nc=6	*low*		*low*		*sustained*		*strong*		*slowest*	*small size*		
**PV2**	**OFF**	BS-On	*No response*											
		WS-Off												
	**ON**	BS-Off	24.6	20.2	272	229	~0%	53	65%	85%	76	180	221	106
		WS-On	17.8	16.4	235	208	~0%	53	79%	81%	73.5		207	
		Nc=12	*low*		*low*		*transient*		*strong*		*med/slow*	*medium/large size*		
**PV1**	**OFF**	BS-On	*No response*, sometimes for D=125um WS-On effect was not completely turned OFF											
		WS-Off												
	**ON**	BS-Off	78	75	270	217	6%	170	20%	21%	107	260	439	156
		WS-On	114	119	272	242	20%	90	28%	42%	86		368	
		Nc=11	*low*		*low*		*sustained*		*weak*		*med/slow*	*large size*		
**PV0**	**OFF**	BS-On	14.4	10.4	152	133	<1%	44	92%	97%	105	199	120	176
		WS-Off	10.0	10.5	127	125	<1%	41	95%	97%	91		120	
	**ON**	BS-Off	14.5	12.5	145	133	1%	52	71%	78%	105	189	219	
		WS-On	12.0	11.5	147	150	1%	48	82%	90%	110		186	
		Nc=13	*low*		*low*		*transient*		*v. strong*		*med/slow*	*medium size*		

S1 Table
